# *Glycyrrhiza* polysaccharide-adjuvanted liposomal vaccine potentiates tumor immunotherapy through lymph node-targeted modulation of the DC-T cell axis

**DOI:** 10.1186/s13046-025-03601-6

**Published:** 2025-11-28

**Authors:** Xiaopan Yao, Keqing Zhang, XiaoKun Zhang, Shengxin Lu, Jinyuan Hu, Yuexuan Wang, Jiayi Lin, Ye Wu, Weidong Zhang, Hongzhuan Chen, Xia Liu, Bei Wang, Xin Luan

**Affiliations:** 1 State Key Laboratory of Discovery and Utilization of Functional Components in Traditional Chinese Medicine, Shanghai Frontiers Science Center of TCM Chemical Biology, Shuguang Lab of Future Health, Institute of Interdisciplinary Integrative Medicine Research and Shuguang Hospital, Shanghai University of Traditional Chinese Medicine, Shanghai, 201203 China; 2 State Key Laboratory for Quality Ensurance and Sustainable Use of Dao-di Herbs, Institute of Medicinal Plant Development, Chinese Academy of Medical Science and Peking Union Medical College, Beijing, 100700 China; 3 School of Pharmacy, Naval Medical University, Shanghai, 200433 China

**Keywords:** Cancer immunotherapy, Vaccine adjuvants, Antigen cross-presentation, Tumor antigen-specific T cells, *Glycyrrhizae* polysaccharides

## Abstract

**Background:**

A key challenge in cancer immunotherapy is that tumor vaccines formulated with conventional aluminum adjuvants often fail to elicit potent cellular immunity and sustained antitumor responses. *Glycyrrhizae* polysaccharides (NGUP), characterized by significant immunomodulation, multi-target antitumor efficacy, and low toxicity, represent promising candidates for next-generation vaccine adjuvants.

**Methods:**

We employed transcriptome analysis, quantitative real-time PCR, and Western blot assays to investigate the mechanism of NGUP in activating bone marrow-derived dendritic cells in vitro. Using confocal microscopy, small animal in vivo imaging, and flow cytometry, we examined the process of tumor antigen-specific T cell response activation by the liposomal vaccine (NGUPL@OVA) in vivo. The efficacy of NGUPL@OVA was evaluated in murine melanoma models (B16-OVA and B16-F10) through immunohistochemistry, immunofluorescence and H&E staining.

**Results:**

NGUP activates dendritic cells through the TLR4/MyD88/TRAF6/NF-κB signaling pathway. NGUPL@OVA demonstrates efficient lymph node targeting capacity, significantly enhancing dendritic cell maturation and antigen cross-presentation, thereby promoting robust CD8^+^ T cell activation and inducing potent cellular immune responses with long-term immunological memory. In both prophylactic and therapeutic settings, NGUPL@OVA exhibits significant melanoma growth inhibition without observable toxic side effects.

**Conclusions:**

NGUP as a novel vaccine adjuvant for cancer immunotherapy effectively overcomes key limitations of conventional aluminum adjuvants, including weak induction of cell-mediated immunity and significant adverse effects, while exhibiting superior immune-stimulating properties.

**Supplementary Information:**

The online version contains supplementary material available at 10.1186/s13046-025-03601-6.

## Introduction

As an important approach in cancer immunotherapy, tumor vaccines activate tumor-specific immune responses to recognize and eliminate tumor cells, offering advantages such as high specificity and low side effects [1, 2]. Currently, two main types have been developed: preventive vaccines including HPV vaccine (Gardasil 9) [3] and HBV vaccine [4], which significantly reduce the incidence of cervical cancer and liver cancer by blocking oncogenic viral infections; and therapeutic vaccines such as the prostate cancer vaccine Sipuleucel-T [5] and melanoma oncolytic viral vaccine T-VEC [6], which have been approved but generally show objective response rates below 30%-40% and are only applicable to specific cancer types. The key limitations affecting their efficacy mainly include weak immunogenicity of tumor antigens, inefficient antigen presentation, immunosuppressive tumor microenvironment, and the lack of highly effective and safe adjuvant systems [7]. These issues not only compromise the clinical efficacy of vaccines but also restrict their widespread application.

Adjuvants are essential elements in vaccines, playing a key role in boosting antigen immunogenicity and regulating immune response mechanisms [8]. Current clinical practice still predominantly employs traditional adjuvants such as aluminum salts [9] (e.g., in HBV vaccine Engerix-B), which primarily function by forming antigen depots and activating the NLRP3 inflammasome, skewing immune responses toward Th2-type humoral immunity while demonstrating limited efficacy in eliciting Th1-type cellular immunity [10]. In contrast, novel adjuvants show superior potential for clinical application. Toll-like receptor (TLR) agonists [11] (e.g., MPL in Cervarix) and cytokines [12] (e.g., GM-CSF in Provenge) can more effectively activate immune responses, with the oncolytic virus T-VEC achieving a 31% objective response rate in melanoma through GM-CSF expression [13]. Although next-generation adjuvants like STING agonists can induce stronger Th1 responses [14], they still face three major challenges: (1) the immunosuppressive tumor microenvironment in solid tumors may compromise adjuvant efficacy; (2) current delivery systems struggle to achieve precise targeting of adjuvants to lymph nodes (LNs) or tumor sites; and (3) potent adjuvants may trigger excessive inflammatory responses while exhibiting issues of high toxicity and cost [15]. Consequently, the development of novel adjuvants that effectively enhance antigen immunogenicity while maintaining favorable safety profiles has become a key research focus in tumor vaccine development.

Polysaccharides, as essential bioactive components of Traditional Chinese Medicine (TCM), exhibit remarkable immunomodulatory and antitumor activities [16, 17]. Their unique advantages in tumor immunotherapy stem from multiple characteristics including multi-target effects, low toxicity, and excellent biocompatibility. These compounds can mimic pathogen-associated molecular patterns (PAMPs) and precisely activate innate immune receptors, such as C-type lectin receptors (CLRs) and Toll-like receptors (TLRs) on antigen-presenting cells [18]. By orchestrating dendritic cell (DC) maturation, T cell priming, and lymphocyte homing, polysaccharides uniquely bridge humoral and cellular immunity [19], effectively addressing current limitations in modern immunotherapy such as low response rates and significant immune-related toxicities. Currently, TCM polysaccharides are commonly used as adjuvant therapies in combination with radiotherapy and chemotherapy for cancer treatment. They exert antitumor effects through multiple mechanisms: activating the immune system, ameliorating the immunosuppressive tumor microenvironment, inducing tumor cell apoptosis, and inhibiting tumor angiogenesis [20]. With stable sources, potent immunomodulatory activity, and favorable biosafety profiles, TCM polysaccharides are considered ideal candidates for vaccine adjuvants [21, 22].

Glycyrrhizae Radix Et Rhizoma has a long history of distinguished clinical application, standing as one of the most extensively and frequently used core medicinal materials in TCM practice for over two millennia [23]. TCM formulations containing Glycyrrhizae Radix et Rhizoma as a principal component, such as Gancao Xiexin Tang and Buzhong Yiqi Tang are commonly used in clinical practice as adjuvant therapies during cancer radiotherapy and chemotherapy. These formulations exhibit immunomodulatory properties, mitigate treatment-related adverse effects, and may contribute to delaying tumor progression [24]. Traditionally, Glycyrrhizae Radix Et Rhizoma is renowned for its spleen-tonifying, qi-replenishing, heat-clearing, detoxifying, and medicinal harmonizing properties. Modern pharmacological studies have identified Glycyrrhiza polysaccharides (GP) as one of the key bioactive constituents responsible for these therapeutic effects [25]. GP primarily consist of monosaccharides including glucose, galactose, arabinose, rhamnose, and mannose [26]. These polysaccharides exhibit a broad molecular weight distribution (10–500 kDa) and contain various glycosidic linkages such as β-(1→3)-D-glucan and α-(1→4)-D-galacturonic acid [27]. This complex chemical architecture endows them with distinctive biological activities. As reported, GP function as potent immunomodulators capable of coordinately regulating both innate and adaptive immune responses [25]. Their immunoregulatory mechanisms include dendritic cell activation [28], macrophage and natural killer (NK) cell stimulation [29], promotion of T/B lymphocyte proliferation, and maintenance of the dynamic balance between T helper 1 (Th1) and T helper 2 (Th2) cells [30]. While GP show promising potential as vaccine adjuvants, several challenges remain in their development into clinically viable tumor vaccine adjuvants: (1) The precise structure-activity relationship between fine structural characteristics of GP and their adjuvant properties has not been fully elucidated; (2) The molecular mechanisms underlying GP-mediated activation of antitumor immune responses require further investigation; and (3) The delivery efficiency and targeting specificity of licorice polysaccharides in vivo need significant improvement.

**Scheme 1:**
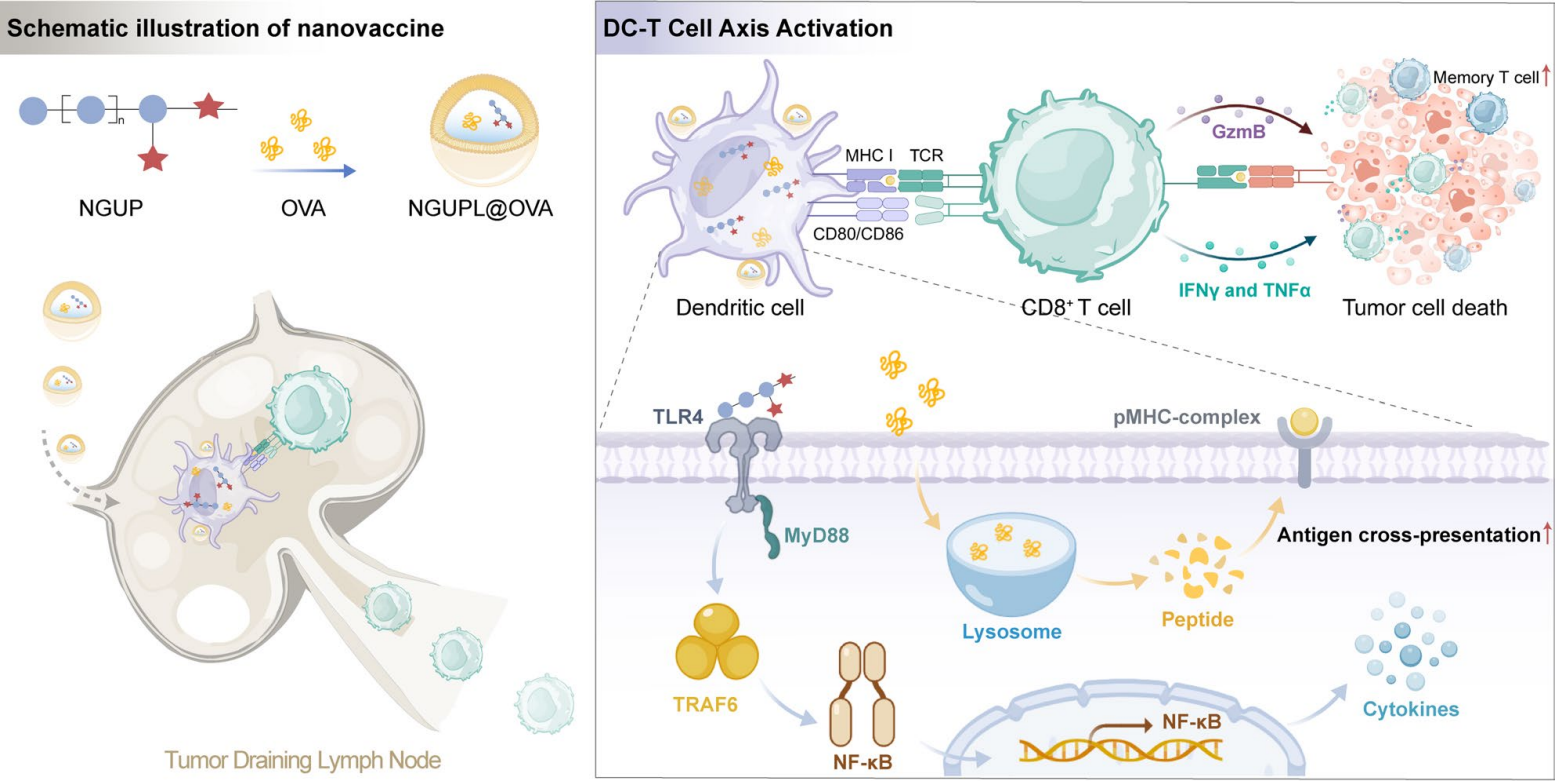
Schematic illustration of the NGUPL@OVA for enhanced cancer immunotherapy. NGUPL@OVA encapsulates polysaccharide and OVA in lipid material. It’s internalized by DCs, enabling antigen cross-presentation and T cell activation. It also induces strong anti-tumor immunity, inhibiting tumor growth

In this study, we isolated a novel polysaccharide (NGUP) from Glycyrrhizae Radix Et Rhizoma and subsequently developed an NGUP-based nanovaccine (NGUPL@OVA) with efficient lymph node-targeting properties based on structural characterization. NGUPL@OVA significantly enhanced DC activation and antigen cross-presentation in mice, leading to robust cellular immune responses and immunological memory. Notably, NGUPL@OVA demonstrated potent antitumor immunity in both prophylactic and therapeutic B16-OVA melanoma models while exhibiting favorable versatility. Mechanistic studies revealed that the NGUP adjuvant possesses excellent biocompatibility and safety profiles. Importantly, we provided the first experimental evidence that NGUP activates DCs through the TLR4/MyD88/TRAF6/NF-κB signaling pathway, thereby potentiating T cell-mediated antitumor immunity. Compared with conventional aluminum-based adjuvants, NGUP addresses two critical limitations - insufficient cellular immune response induction and significant adverse effects - positioning it as a highly promising vaccine adjuvant candidate. These findings provide preliminary but important data for the development of novel vaccine adjuvants with enhanced efficacy and reduced toxicity. The study not only advances our understanding of polysaccharides as immunomodulators but also offers new possibilities for cancer immunotherapy through vaccine development (Scheme 1).

## Materials and methods

### Materials and reagents

All reagents and solvents were purchased from Adamas-beta, Energy Chemical, GL Biotech, or Sinopharm Chemical Reagent Co. Ltd. The Glycyrrhizae Radix Et Rhizoma were purchased from Bozhou Baopu Pharmaceutical Co., Ltd. (Bozhou City, Anhui Province, China). DEAE-52 cellulose was supplied by Beijing Solarbio Science & Technology Co., Ltd. (Beijing, China). Sephadex G-100 was obtained from GE Healthcare (Little Chalfont, UK). D-Glucose was provided by the National Institutes for Food and Drug Control (NIFDC, Beijing, China). Dialysis bags (molecular weight cutoff: 3.5 kDa) were purchased from Shanghai Yuanye Bio-Technology Co., Ltd. (Shanghai, China).

### Cell culture and animals

The B16-OVA cell line was obtained from Zhejiang Meisen Cell Technology Co., Ltd (Pan’an, Zhejiang, China) and cultured in complete Dulbecco’s Modified Eagle’s Medium (DMEM). The B16F10 cell line was purchased from the Cell Bank of the Chinese Academy of Sciences (Shanghai, China) and cultured in complete Dulbecco’s Modified Eagle’s Medium (DMEM). The complete medium was mixed with 10% fetal bovine serum (FBS, Gibco, 10091148) and 1% penicillin-streptomycin (HyClone, SV30010). Bone marrow derived dendritic cells (BMDCs) were isolated from 6-week-old C57BL/6 female mice. Briefly, after the tibias and femurs of the mice were collected in a sterile environment, the bone marrow cells were flushed with RPMI 1640. The suspension was filtered through a 70 μm cell filter to remove the tissues and fragments. The RBCs were split with a RBC lysis buffer. After centrifugation at 1200 rpm for 5 min, the monocytes were washed and counted. The BMDCs were cultured in a large dish at 5 × 10^6^ cells/dish density with complete RPMI 1640 containing GM-CSF (20 ng mL^− 1^). All the cell lines were maintained in a humidified incubator (Thermo Fisher, USA) at 37℃ with 5% CO_2_. All the animals were cared and treated according to the instructions and approval of the Institutional Animal Care and Use Committee of Shanghai Model Organisms Center, Inc (IACUC NO. 2025-0020). Female C57BL/6 mice (4–6 weeks old) was purchased from Shanghai Model Organisms Center, Inc.

### Preparation and purification of NGUP

The dried *Glycyrrhiza uralensis* slices were extracted three times with boiling water, each time for 1 h. The extracted solutions were concentrated and precipitated by 80% ethanol for 12 h. The precipitate was freeze-dried to obtain the crude polysaccharide extract. Subsequently, the crude extract was deproteinized using a cation exchange resin, dialyzed (molecular weight = 3.5 kDa) to remove low-molecular-weight impurities, and freeze-dried. The resulting material was further fractionated via a DEAE-52 cellulose column using stepwise gradients of ultrapure water, 0.5 M NaCl, and 1.0 M NaCl for elution. The major elution peak fractions were collected and freeze-dried, yielding the major fraction as NGUP. Finally, NGUP was further purified through a Sephadex G-100 dextran gel column, and the major peak fractions were collected, concentrated, and freeze-dried to obtain homogeneous polysaccharides for structural characterization.

### Structure elucidation of NGUP

The homogeneity and molecular weight distribution of NGUP were analyzed by high-performance gel permeation chromatography (HPGPC). Chromatographic Conditions: Column: TSK-GEL-GMPWXL; mobile phase: 0.1 M NaNO_3_ containing 0.05% (w/v) NaN_3_ in ultrapure water; flow rate: 0.6 mL/min; injection volume: 20 µL; column temperature: 35 °C; detector: refractive index detector.

Determination of Monosaccharide Composition of NGUP by Ion Chromatography (IC). Chromatographic Conditions: Column: Dionex Carbopac™ PA20; mobile phase: A: H_2_O, B: 15 mM NaOH and 15 mM NaOH + 100 mM NaOAc; flow rate: 0.3 mL/min; injection volume: 5 µL; column temperature: 30 °C; detector: electrochemical detector.

Glycosidic linkage analysis was performed via methylation-gas chromatography-mass spectrometry (GC/MS): Complete methylation (verified by FT-IR) was followed by TFA hydrolysis (2 M, 90 min), NaBH₄ reduction, and acetylation; derivatives were separated on a Rxi^®^-5Sil MS column (3 °C/min to 250 °C).

Structural elucidation was completed by nuclear magnetic resonance (NMR) spectroscopy (600 MHz, D₂O): ¹H/¹³C, COSY, HSQC, and HMBC spectra were acquired to resolve sugar sequences and anomeric configurations.

### Synthesis and characterization of NGUPL@OVA

“BL” refers to blank liposomes; “BL@OVA” denotes OVA-loaded liposomes; “NGUPL” indicates NGUP-loaded liposomes; and “NGUPL@OVA” represents liposomes co-loaded with OVA and NGUP. BL and NGUPL were synthesized via reverse-phase evaporation: soybean phospholipid, cholesterol, and Tween-80 were dissolved in chloroform and diethyl ether, while NGUP was dissolved in phosphate-buffered saline (PBS, pH 7.3). The BL was prepared without the addition of NGUP. The organic and aqueous phases were emulsified by ice-bath ultrasonication to form a stable water-in-oil emulsion. After rotary evaporation to a gel state, PBS was added for 15-minute rehydration, followed by 20-minute homogenization using an ultrasonic cell disruptor. Finally, the liposome was filtered sequentially through 0.45 μm and 0.22 μm Millipore membranes to ensure sterility and then stored at 4 °C for subsequent experiments. OVA encapsulation employed a triple freeze-thaw cycle: NGUPL-OVA or BL-OVA mixtures (10:1 v/v, 1 mL NGUPL or BL + 0.1 mL OVA) underwent freezing (-20 °C, 12 h) and thawing (37 °C, 100 rpm, 30 min) repeated three times to obtain NGUPL@OVA and BL@OVA, respectively. The entrapment efficiency (EE) of NGUP was analyzed using a Sephadex G-50 mini-column centrifugation method: Liposomal suspensions were loaded onto columns and centrifuged (1,500 rpm, 5 min) to separate free NGUP. Following liposome disruption with 10% Triton X-100, total NGUP and encapsulated NGUP were quantified via the phenol-sulfuric acid method, with EE% calculated as: EE% = (Encapsulated NGUP / Total NGUP) × 100%. For OVA, entrapment efficiency was determined by ultracentrifugation: Liposome were centrifuged (12,000 rpm, 1 h), and the supernatant was collected to measure free protein content (Cs) using a BCA Protein Assay Kit. Total protein content (Ct) was measured after liposome disruption, with EE% calculated as: EE% = (1 - Cs/Ct) × 100%. The drug loading capacity (W%) was calculated using the formula: W% = [(Total mass of encapsulated NGUP and OVA) / (Total mass of membrane components including soybean phospholipid and cholesterol)] × 100%. Characterization included transmission electron microscope (TEM) analysis (Hitachi, 120 kV), dynamic light scattering (DLS) for hydrodynamic size, and laser doppler electrophoresis for zeta potential (Zetasizer Nano ZS, Malvern).

### BMDCs maturation

The BMDCs were extracted from C57BL/6 mice. The bones of mice were soaked in PBS containing 100 U/mL penicillin and streptomycin, and the bone marrow cells were collected via flushing the femurs and tibias with RPMI 1640 medium. The cells were cultured in the RPMI 1640 medium containing 10% FBS and recombinant murine GM-CSF (20 ng/mL) for 6 days to differentiate into immature BMDCs. On day 7, the immature BMDCs were treated with NGUP (0.5/5/50 µg/ml) or free OVA, BL@OVA, NGUPL or NGUPL@OVA (NGUP, 50 µg/ml and OVA, 10 µg/ml) for 24 h. BMDCs incubated with 1 µg/ml lipopolysaccharide (LPS) were used as a positive control. Then, the cells were collected and centrifuged at 400 ×g for 5 min at 4℃. The supernatant was collected separately for analysis via an ELISA kit to quantify the secretion of the cytokines IL-6, TNF-α and IL12p70. 3% BSA was used to block the nonspecific binding sites on the cells for 15 min in an ice bath. Then, the cells were washed with PBS and staining buffer containing anti-Mouse CD11c-PE-Cy7 (BD, 558079), anti-Mouse CD80-APC (BD, 560016), anti-Mouse CD86-PE (BD, 553692), and anti-Mouse MHCII (BD,562009) was added for 30 min incubation in an ice bath. Finally, after being washed with PBS, the cells were resuspended in staining buffer and analyzed by Beckman Coulter CytoFLEX LX flow cytometer (Beckman Coulter, USA).

### Cellular uptake of OVA-FITC in vitro

BMDCs were seeded in the 20 mm diameter confocal dish and cultured overnight at a density of 5 × 10^4^ cells per well. The cells were exposed to free OVA, NGUP + OVA, BL@OVA, or NGUPL@OVA containing a final fluorescein isothiocyanate (FITC) labeled OVA concentration of 10 µg/mL at 37 °C for 24 h. The lysosomes were stained by Lyso-Tracker Red (5 µM) for 25 min, and the cell nuclei were stained with Hoechst 33,342 (5 µM) (Ex/Em = 350/461 nm) for 5 min. A GE DeltaVision OMX SR was used for imaging. For Flow Cytometry (FCM) analysis, cells were seeded in a 12-well plate at 1 × 10^6^ cells/well density. After being treated with different treatments, the cells were collected and transferred into a tube for centrifugation at 400 ×g for 5 min. After being washed with PBS, the cells were analyzed via FCM.

### Antigen presentation assay

BMDCs were incubated in glass bottom dishes with preloaded rat tail collagen at a density of 1 × 10^5^ cells per well and cultured overnight. They were treated in different groups for 24 h. After being washed with PBS, the cell membranes were stained with DiO (5 µM) for 20 min. 4% paraformaldehyde was added to fix the cells for 20 min incubation, and 1% BSA was used to block the nonspecific binding sites for 1 h. Then, the cells were stained with PE anti-mouse H2K^b^ bound to the SIINFEKL (Biolegend, 141603, 1:100) and Hoechst (5µM) for imaging. For FCM, BMDCs were seeded in a 12-well plate at a density of 1 × 10^5^ cells per well. After 24 h incubation, cells were collected and centrifuged for blocking with 3% BSA. The SIINFEKL-H2K^b^ conjugated PE was used for staining for 30 min. After being washed with PBS, the cells were analyzed by FCM.

### In vitro T-cell activation

Splenocytes were isolated from the spleens of 6-week-old C57BL/6 mice via lymphocyte density gradient centrifugation according to the Ficoll-Paque PREMIUM (density: 1.084 ± 0.001 g/mL; Cytiva, 17544602). The above-treated BMDCs were collected and coincubated with spleen lymphocytes at a ratio of 1:5 for 24 h. The cells were collected and centrifuged at 400 ×g for 5 min at 4℃. The cells were stained with anti-Mouse CD3-PE (BD, 553240), anti-Mouse CD8a-APC (Biogems, 10112-80-100), anti-Mouse CD4-PerCP-Cy5.5 (BD, 550954), and anti-Mouse CD69-PE-Cy7 (BD, 552879) according to the FCM instructions. Then, the above-treated T cells were added to B16-OVA tumor cells for a further 24 h. The tumor cells were stained with crystal violet for observation of cell viability. The release of LDH was measured via a cytotoxicity LDH release assay Kit-WST (DOJINDO, CK-12). The cells were stained with anti-Mouse CD3-PE (BD, 553240), anti-Mouse CD8a-APC (Biogems, 10112-80-100), anti-Mouse CD4-PerCP-Cy5.5 (BD, 550954), and anti-Mouse Granzyme B (eBioscience 11-8898-82) according to the FCM instructions.

### RNA-Seq of NGUP on BMDCs

BMDCs were seeded at the density of 5 × 10^6^ cells per dish with medium containing granulocyte-macrophage colony-stimulating factor (GM-CSF) (20 ng mL^− 1^). They were treated with NGUP (50 µg/mL) for 24 h, using untreated one as negative control. Then, the cells were wash and added 1 mL Trizol for collection. The subsequent analysis was conducted by Oebiotech (Shanghai, China).

### Real time PCR

BMDCs cells were seeded at 2.0 × 10^6^ cells/well in a 6 well plate overnight. Then the cells were incubated for 24 h with NGUP (50 µg/mL) and LPS (1 µg/mL) respectively. After the incubation, total RNA was extracted using the RNAiso Plus (TaKaRa, Bio, Inc) reagent according to the manufacturer’s protocol. Real-time PCR was employed for the determination of TLR4, MyD88, TRAF6 and NF-κB mRNA levels. The isolated RNA was re-suspended in 30 µL diethyl-pyrocarbonate water, quantified by measurement for the absorbance ratio at 260/280 nm, and then stored at -70℃ prior to cDNA synthesis. First-strand cDNA was synthesized from 1 µg of total RNA using Oligo dT primers and M-MLV reverse transcriptase (TaKaRa, Bio, Inc) according to the manufacturer’s instructions. Real-time PCR was performed on an ABI PRISM 7300 Detection System (Applied Biosystems, USA). A total 20 µL of the reaction mixture containing 10 µL of 2 × SYBR Green I PCR Master Mix (TaKaRa, Bio, Inc), 2 µL of cDNA, 1 µL of each primer (10 µM) and 0.5 µL of ROX Reference Dye (50 ×), 5.5 µL RNnase-free H_2_O. The following thermal cycling conditions were used: a single step at 95 °C for 30 s, followed by 40 cycles of 95 °C for 5 s and 60 °C for 34 s A dissociation curve was run for each plate to confirm that a single PCR product was formed. A non-template reaction served as the negative control. The relative mRNA levels were determined using the Δ cycle threshold (ΔCt) method with β-actin serving as a reference gene. For each of the target genes, the ΔΔCt values of all the samples were calculated by subtracting the average ΔCt of the control group from the average ΔCt of the experiment group. The ΔΔCt values were converted to fold differences by raising 2 to the power of − ΔΔCt (i.e., 2^−ΔΔCt^).

### Western blot assay

In Toll-like 4 pathway activation verification of NGUP, BMDCs were treated with NGUP (50 µg/mL) for 24 h. Then cells were washed with cold PBS three times and lysed in cold NP40 buffer containing protease inhibitors and phosphatase inhibitors. The lysed cells were collected and centrifuged at 15,000 rpm for 15 min at 4℃. The supernatants were collected and quantified with a BCA assay kit (Beyotime, P0010S). All the samples were diluted with loading buffer and separated in 12.5% SDS-PAGE. These samples were transferred onto polyvinylidene difluoride (PVDF) membranes (GE, 10600021) and blocked in Tris-buffered saline containing 1% Tween-20 (TBST, pH 7.4) and 5% skim milk for 1 h. The corresponding molecular weight of the film was cut and incubated with primary antibodies (anti-TLR4 Rabbit pAb, MyD88 Recombinant Rabbit mAb, TRAF6 Recombinant Rabbit mAb, Phospho-NF-κB p65) respectively at 4℃ overnight. The movies were washed with TBST for 10 min three times and then incubated with secondary antibodies for 1 h. Detection was performed via a gel imaging system (Tanon, China), and the bands were analyzed using Image J software. β-actin was used as an internal control.

### In vivo distribution

OVA was conjugated to the Cy5.5 fluorophore via a thiol-maleimide reaction. The conjugation was performed by incubating OVA-Ahx-cys with Cy5.5-maleimide (Xi’an Ruixi Biological Technology Co., Ltd.) in PBS, followed by lyophilization. The NGUP-Cy7 conjugate was synthesized via a two-step procedure. Briefly, primary amine groups were introduced to the polysaccharide by reductive amination with hexamethylenediamine, and the successful modification was confirmed by a positive ninhydrin assay. The aminated NGUP was then reacted with Cy7-NHS ester in PBS (pH 8.0) under light-protected conditions. The final conjugate was purified through repeated ethanol precipitation and collected by lyophilization. Cy5.5 fluorescently labeled OVA-Cy5.5 and Cy7 fluorescently labeled Cy7-NGUPL@OVA-Cy5.5 were intradermally injected into mice to study the biodistribution and antigen release kinetics in vivo. The fluorescence signal was captured with an IVIS Spectrum system (VISQUE In Vivo Elite, VISQUE, Korea) at different time points (*n* = 3). Subsequently, Inguinal Lymph Nodes (ILNs) and Axillary Lymph Nodes (ALNs) were isolated at 2 h, 4 h, and 8 h for ex vivo imaging (*n* = 3), followed by the implantation of LNs into frozen sections. The sections were then stained with DAPI and anti-CD11c-FITC and imaged using a Leica TCS SP8 confocal laser scanning microscopy (CLSM; Leica Microsystems, Germany). Additionally, three ILNs and ALNs were prepared as cell suspensions for FCM analysis.

### Immunization strategy

Six groups of female C57BL/6 mice (6–8 weeks old) mice were randomly assigned, including control, OVA (20 µg OVA), BL@OVA (20 µg OVA), NGUP (high) + OVA (200 µg NGUP and 20 µg OVA), NGUPL (low) @OVA (50 µg NGUP and 20 µg OVA), NGUPL (high) @OVA (200 µg NGUP and 20 µg OVA) and AL + OVA (25 µg Al and 20 µg OVA). On days 0 ,7 and 14, the mice were injected intradermally with the vaccines. Lymphatic node is collected on days 3 and 5 to evaluate DC cell maturation and antigen cross-presentation. On day 7, after vaccination of C57BL/6 N mice, the spleens were isolated and dissociated into single-cell suspensions. Splenocytes were inoculated in 6-well plate at a density of 3 × 10^6^ cells per well. The samples were incubated with OVA_257 − 264_ at a concentration of 10 µg/mL for 24 h. Then the proportion of OVA-specific T cells was analyzed via FCM. Spleen is collected on day 21 to evaluate immune memory. The cells were stained with anti-Mouse CD45-PerCP (Biolegend, 103130), anti-Mouse CD3-APC-Cy7 (BD, 553240), anti-Mouse CD8a-APC (Biogems, 10112-80-100), anti-Mouse CD4-FITC (BD, 550954), anti-Mouse CD44-PE (BD, 559250) and CD62L-BV421 (BD, 562910) according to the FCM instructions.

### Prophylactic and therapeutic efficacy in melanoma-bearing mice

Female C57BL/6 mice (6–8 weeks old) were purchased from Shanghai Model Organisms Center, Inc. and kept under SPF conditions. The animal experiment in this study was approved by the Committee on the Ethics of Animal Experimentation of the Shanghai Model Organisms Center, Inc. For the prophylactic study, different vaccines were intradermally injected at the tail base into the female C57BL/6 mice of each group at oneweek intervals three times. Seven days after the final vaccination, the vaccinated mice were subcutaneously challenged with 5 × 10^5^ B16-OVA or 5 × 10^5^ B16F10 cells. The tumor volume was monitored daily via a vernier calliper and calculated via the formula (volume= (length×width^2^)/2). For the therapeutic study, female C57BL/6 mice (6 weeks old) were subcutaneously injected with 5 × 10^5^ B16-OVA or 5 × 10^5^ B16F10 cells. Female C57BL/6 mice were intradermally vaccinated with different kinds of vaccines, as described in the main text, on days 3, 7 and 11. The tumor volume was monitored daily via a vernier calliper and calculated via the formula (volume= (length×width^2^)/2). At the end of the experiment, 5 animals in each group were euthanized, and their blood was collected for blood panel analysis. Tumors were collected, fixed in 4% paraformaldehyde, and subjected to H&E (histopathology), Ki-67 (proliferation), and TUNEL (apoptosis) staining according to standard protocols. Tumor Suppression Efficiency (%) = (1- Mean tumor volume in treatment group/ Mean tumor volume in control group) × 100.

### Analysis of immune cells in lymph node, spleen and tumor

For immune cell analysis, lymph node tissues were ground and washed with DMEM to obtain single-cell suspensions. Tumor tissues were harvested and digested with a Tumor Dissociation Kit (Miltenyi, 130-096-730). Lymph node cells suspensions and tumor cells suspensions were divided into two parts, blocked with 3% BSA for 20 min and stained with anti-Mouse CD45-FITC, anti-Mouse CD11c-PE-Cy7, anti-Mouse CD80-APC, anti-Mouse CD86-BV421, and PE anti-mouse H2K^b^ bound to SIINFEKL for DC maturation, anti-Mouse CD45-PerCP (Biolegend, 103130), anti-Mouse CD3-PE (BD, 553240), and anti-Mouse CD8a-APC (Biogems, 10112-80-100) for T-cell infiltration. Splenocytes were isolated from the spleen using the same dissociation protocol and stained identically for T-cell infiltration analysis.

### Statistical analysis

Data were shown as the mean ± standard deviation (S.D.). Statistical analysis was performed with ANOVA with Tukey’s multiple comparison test by GraphPad Prism 8.0. * and # indicated significant differences: *p < 0.05, **p < 0.01, and ***p < 0.001 vs. Control; #*p* < 0.05, ##*p* < 0.01, and ###*p* < 0.001.

## Results and discussion

### Purification and structural elucidation of NGUP

The crude polysaccharide was extracted from TCM Glycyrrhizae Radix Et Rhizoma using aqueous extraction followed by ethanol precipitation. Further purification was performed to remove proteins and small-molecule components (Mw < 3.5 kDa) from the crude polysaccharide by resin adsorption and dialysis. Subsequently, the product was repeatedly separated via DEAE-cellulose and Sephadex gel column chromatography, yielding a novel neutral polysaccharide designated as NGUP. The chemical composition of NGUP was determined through the phenol-sulfuric acid method and BCA assay, revealing a total carbohydrate content of 96.40% with negligible amounts of protein and nucleic acid impurities.

The mass-average molar mass (Mw) of NGUP was estimated to be 14.68 kDa and the number-average molecular weight (Mn) was 9,352 Da by HPGPC. The chromatograms of NGUP (Fig. S1a-b) displayed a single symmetrical peak with a polydispersity index (α) of 1.57, confirming the homogeneity of NGUP. IC analysis demonstrated that NGUP was predominantly composed of glucose (80.88%) with arabinose (14.30%) and galactose (4.82%), as shown in Fig. S1c-d. The results from methylation analysis, GC/MS (Table S1), and NMR spectroscopy (Fig. S2-7) revealed that NGUP is a branched heteropolysaccharide [31]. Its primary structural unit might consist of a backbone formed by the linkage of →4)-α-D-Glcp-(1→ along with minor amounts of →5)-α-L-Araf-(1→ and →4,6)-β-D-Glcp-(1→. The side chains are mainly composed of α-D-Glcp-(1→ linked to the O-4 position of the →4)-α-D-Glcp-(1→ residue and α-L-Araf-(1→ attached to the O-6 position of the →4,6)-β-D-Glcp-(1→ residue. The chemical structure of NGUP is showed in Fig. S8, with its corresponding chemical shift assignments detailed in Table S2.

### Activation effects and mechanisms of NGUP on BMDCs in vitro

**Fig. 1:**
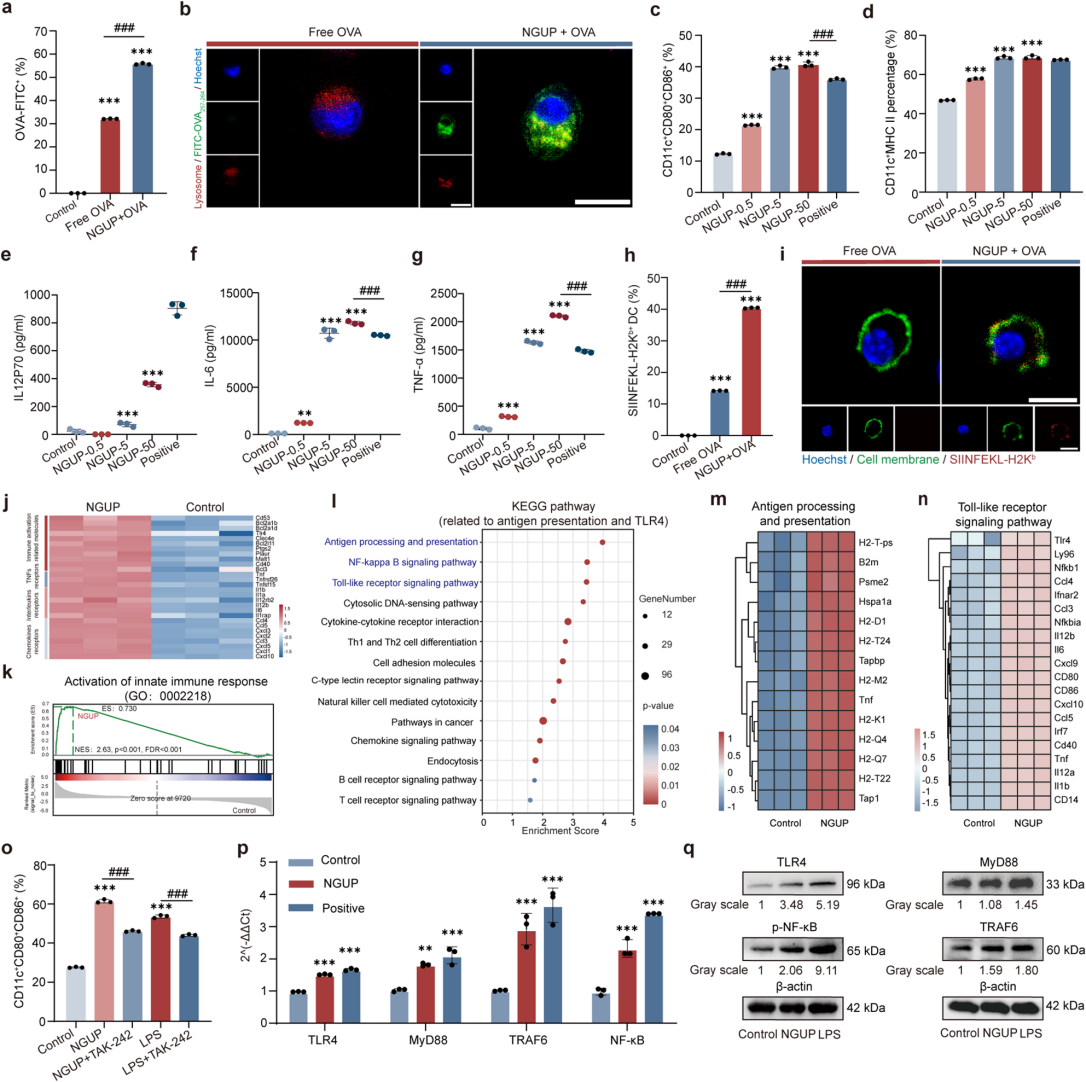
Activation effects and mechanisms of NGUP on BMDCs in vitro. **a** OVA-FITC in BMDCs was detected and quantified via FCM. **b** The intracellular uptake of FITC-OVA in DC cells was examined via CLSM after treatment with free FITC-OVA or NGUP + OVA. Lysosomes (Lyso-Tracker Red, red); cell nucleus (Hoechst 33342, blue) (scale bar, 10 μm). **c** The expression of CD80 and CD86 in BMDCs was measured by FCM. **d** The expression of MHC II in BMDCs was measured by FCM. **e-g** Levels of IL12P70, IL-6 and TNF-α at cell supernatantwere analyzed by ELISA. **h** PE-conjugated SIINFEKL-H2K^b^ in BMDCs was detected and quantified via FCM. **i** The SIINFEKL-H2K^b^ expressed on the surface of BMDCs was examined via CLSM after treatment with free OVA and NGUP. SIINFEKL-H2K^b^ (PE-conjugated SIINFEKL-H2K^b^, red); cell membrane (DiO, green); cell nucleus (Hoechst 33342, blue) (scale bar, 10 μm). **j** Cluster heatmap showing the transcript expression ofrelated genes. **k** GSEA analysis of activation of innate immune response (GO:0002218) from GO enrichment. **l** KEGG pathway analysis from RNA-Seq of BMDCs treated by NGUP. **m** Related upregulated differential genes of antigen processing and presentation compared NGUP to Control group displayed by heat map. **n** Related upregulated differential genes of Toll-like receptor signaling pathway compared NGUP to Control group displayed by heat map. **o** CD80 and CD86 expression in BMDCs after treated by NGUP or LPS with TAK-242 detected by FCM. **p** TLR4, TRAF6, MyD88, phospho-NF-κB mRNA abundance in BMDCs was assessed via qPCR analysis following treatment with NGUP and LPS. **q** TLR4, TRAF6, MyD88, phospho-NF-κB protein expression in BMDCs was assessed via Western blot analysis following treatment with NGUP and LPS. The gating strategy for FCM is shown in Fig. S12. Data are shown as mean ± S.D. (*n* = 3). Statistical significance: *P < 0.05, ** *P* < 0.01 and *** *P* < 0.001 vs. Control; # *P* < 0.05, ## *P* < 0.01 and ### *P* < 0.001

To evaluate the bioactivity of NGUP, we examined its stimulatory effects on BMDCs in vitro. CCK-8 assays demonstrated that NGUP exerted no significant effect on dendritic cell viability (Fig. S9a). OVA was labeled with FITC to facilitate intracellular tracking. FCM (Fig. 1a) analysis and CLSM (Fig. 1b) images revealed that, NGUP significantly enhanced the uptake of OVA by BMDCs compared to Free OVA, and exhibited a time-dependent pattern within 24 h (Fig. S9b). Additionally, FCM results demonstrated that, NGUP significantly upregulated the expression of DC maturation markers CD80 and CD86 (Fig. 1c) as well as MHC II (Fig. 1d) in a dose-dependent manner, compared with the control group. Mature DCs secrete various cytokines to exert their functions [32, 33]. We quantified the levels of IL-12p70 (Fig. 1e), TNF-α (Fig. 1f), and IL-6 (Fig. 1g) in DC supernatants using ELISA, and the trends were consistent with the detection results of DCs maturation markers. These findings collectively indicate that NGUP can significantly promote the maturation of BMDCs. Cross-presentation of antigens by DCs is one of their unique functions [34]. The processed antigen peptides bind to MHC I and are presented on the cell surface for recognition by CD8⁺T cells [35]. SIINFEKL-H2Kᵇ is a classic antigen peptide-MHC I complex [36]. FCM analysis demonstrated that NGUP significantly enhanced SIINFEKL-H2Kᵇ expression compared to the free OVA group (Fig. 1h). CLSM images provided more direct visual evidence, showing markedly increased expression of the SIINFEKL-H2K^b^ on the surface of NGUP-treated BMDCs (Fig. 1i). These results collectively indicate that NGUP could effectively promote antigen capture and cross-presentation by DCs. In addition, NGUP also exhibits the function of promoting T cell and macrophage functions (Fig. S9c-j).

To elucidate the mechanism underlying NGUP-induced DCs activation, we performed RNA sequencing (RNA-seq) to analyze transcriptomic changes in BMDCs treated with NGUP compared with PBS-treated controls. The cluster heatmap of differentially expressed genes (Fig. 1j) revealed that these genes were predominantly associated with immune activation-related molecules, including tumor necrosis factors (TNF) and receptors, interleukins and receptors, as well as chemokines and receptors. Gene Set Enrichment Analysis (GSEA) revealed significant activation of the “activation of innate immune response” in NGUP-treated cells (ES = 0.730, NES = 2.63, *p* < 0.001), indicating that the differentially expressed genes were primarily enriched in biological processes related to innate immunity (Fig. 1k). Kyoto Encyclopedia of Genes and Genomes (KEGG) pathway analysis further identified “antigen processing and presentation” and “Toll-like receptor signaling pathway” as key pathways, highlighting their central role in NGUP response (Fig. 1l). Genes that are associated with the “antigen processing and presentation” pathway are presented in Fig. 1m. Furthermore, we identified significantly upregulated genes, including the maturation markers CD80 and CD86 (Fig. 1n), which were highly expressed in previous DCs maturation experiments (Fig. 1c). Additionally, the downstream cytokine genes IL-6 and IL-12 in the TLR4 signaling pathway showed significant activation (Fig. 1n), which corroborated well with the elevated cytokine levels measured by ELISA assays (Fig. 1e-f). It is reported that polysaccharides have the similar mechanism with LPS to activate DCs via TLR4-MyD88 signaling pathway [37, 38]. To investigate the role of TLR4 in NGUP-induced DCs maturation, BMDCs were pretreated with the TLR4-specific inhibitor TAK-242 4 h prior to NGUP exposure, with LPS serving as a positive control for TLR4 activation. FCM analysis demonstrated that TAK-242 pretreatment significantly downregulated CD80/CD86 expression and markedly inhibited the activation levels in LPS-treated groups, confirming the pivotal regulatory role of TLR4 signaling (Fig. 1o). Subsequent qPCR (Fig. 1p) and Western blot analyses (Fig. 1q) provided complementary validation at both transcriptional and protein levels. In conclusion, both NGUP and LPS treatments significantly upregulated the expression of TLR4 and its downstream signaling molecules (MyD88 and TRAF6), while concurrently enhancing phosphorylation of the NF-κB p65 subunit, thereby comprehensively verifying the activation cascade of the TLR4/MyD88/TRAF6/NF-κB signaling pathway.

However, natural products often exhibit multi‑target characteristics [39]. In this study, KEGG enrichment analysis also revealed that NGUP upregulates pathways related to cytokine receptors and C-type lectin receptors (Fig. 1l). In addition, the TLR4 signaling pathway does not function in isolation but engages in crosstalk with other immune signaling pathways. It has been documented that *Glycyrrhiza* Polysaccharide can exert its effects by regulating the MAPK/PI3K/AKT pathway [40]. Thus, while our data establish the TLR4/MyD88/TRAF6/NF‑κB axis as the primary signaling route for NGUP, future studies employing more comprehensive approaches will be needed to explore the potential roles of these parallel pathways.

#### Synthesis and characterization of NGUPL@OVA

**Fig. 2:**
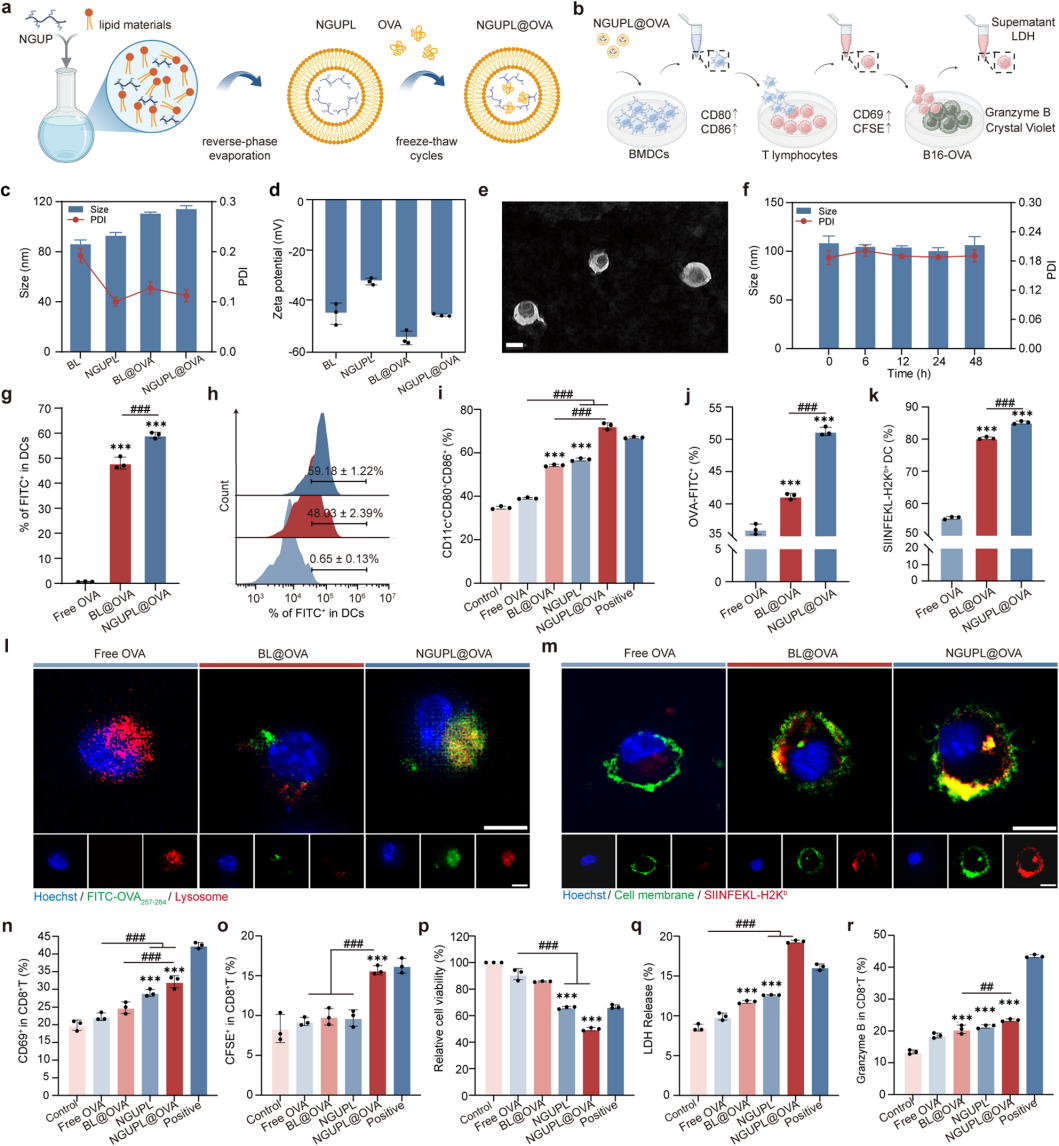
Synthesis, characterization and anti-tumor immunological activity of NGUPL@OVA. **a** Schematic illustration to show the preparation of NGUPL@OVA nanovaccines. **b** Schematic diagram of T-lymphocyte activation and tumor cell killing facilitated by BMDCs treated with NGUPL@OVA nanovaccines. **c**,** d** Particle size, zeta potentials and PDI measured by DLS. **e** TEM (Scale bar = 100 nm) of NGUP@OVA. **f** Size stability from aqueous solutions in 10% FBS of NGUP@OVA. Statistical data (**g**) and representative flow cytometry diagram (**h**) showing the FITC signals of BMDC cells after incubation with different nanoparticle. **i** CD80 and CD86 expression in BMDCs after treatment with Free OVA, BL@OVA, NGUPL, NGUPL@OVA and LPS. **j** OVA-FITC in BMDCs was detected and quantified via FCM. **k** PE-conjugated SIINFEKL-H2K^b^ in BMDCs was detected and quantified via FCM. **l** The intracellular uptake of FITC-OVA in DC cells was examined via CLSM after treatment with free FITC-OVA, BL@OVA or NGUPL@OVA. Lysosomes (Lyso-Tracker Red, red); cell nucleus (Hoechst 33342, blue) (scale bar, 10 μm). **m** The SIINFEKL-H2K^b^ expressed on the surface of BMDCs was examined via CLSM after treatment with free OVA, BL@OVA and NGUPL@OVA. SIINFEKL-H2K^b^ (PE-conjugated SIINFEKL-H2K^b^, red); cell membrane (DiO, green); cell nucleus (Hoechst 33342, blue) (scale bar, 10 μm). **n** FCM analysis of the percentages of CD69^+^T lymphocytes in the co-incubation system. **o** FCM analysis of the percentages of CFSE^+^T lymphocytes in the co-incubation system. **p** Quantification of tumor cell viability by crystal violet staining after 24 h co-culture with T cells. **q** Detect LDH release in the supernatant of the co-culture system. **r** FCM analysis of the percentages of Granzyme B^+^T lymphocytes in the co-incubation system. The gating strategy for FCM is shown in Fig. S12. Data are shown as mean ± S.D. (*n* = 3). Statistical significance: *P < 0.05, ** *P* < 0.01 and *** *P* < 0.001 vs. Control; # *P* < 0.05, ## *P* < 0.01 and ### *P* < 0.001

The above results demonstrated NGUP’s potent immunostimulatory effects through BMDCs activation, leading us to hypothesize its potential as a tumor vaccine adjuvant for enhanced cancer immunotherapy. Based on this premise, we developed the nanovaccine NGUPL@OVA by co-encapsulating NGUP and OVA within liposomal formulation for subsequent investigations. NGUPL was fabricated via reverse-phase evaporation of NGUP and lipids, with OVA subsequently loaded by three freeze-thaw cycles (Fig. 2a). NGUPL exhibited favorable encapsulation efficiency (82.73 ± 1.39%) and drug loading capacity (5.03 ± 0.38%) for OVA. DLS analysis revealed that NGUPL@OVA had an average hydrodynamic diameter of 104.53 ± 2.81 nm with a PDI of 0.16 ± 0.04 and a zeta potential of -40 ± 0.47 mV (Fig. 2c-d). This particle size falls within the optimal range (20–200 nm) for passive drainage to lymph nodes (LNs) [41]. TEM images demonstrated that NGUPL@OVA nanoparticles exhibited spherical morphology with uniform size, well-defined circular shape, and no apparent aggregation (Fig. 2e).

The release profile of OVA from NGUPL@OVA are shown in Fig. S10a. A rapid release phase was observed within the first 6 h, followed by a sustained slow-release phase, achieving a cumulative release rate of 46.69%. NGUP exhibits a lower (13.20%) and more gradual release kinetic profile compared to OVA (Fig. S10b), attributable to its larger molecular size. Further studies demonstrated that NGUPL@OVA exhibited excellent stability in physiological environments (Fig. 2f), showing no phase separation or precipitation within 48 h while maintaining stable particle size, PDI, and zeta potential (Fig. S10c-d). Furthermore, NGUPL@OVA could be efficiently internalized by BMDCs according to the FCM (Fig. 2g-h) and CLSM results (Fig. S10e). In summary, this nanovaccine demonstrates excellent stability and can be efficiently internalized by DCs, enabling controlled and sustained antigen release.

### Anti-tumor immunological activity of NGUPL@OVA in vitro

We next evaluated the immunomodulatory effects of NGUPL@OVA on immune cells in vitro, with the experimental scheme designed as shown in Fig. 2b. CLSM imaging revealed that NGUPL@OVA significantly enhanced OVA uptake by BMDCs compared to BL@OVA (Fig. 2l), demonstrating a time-dependent pattern within 24 h (Fig. S11a), which was corroborated by FCM analysis (Fig. 2j). The differential uptake efficiency between NGUPL@OVA and BL@OVA by BMDC suggests possible variations in the physicochemical properties of these liposomes or in their recognition mechanisms by cells. Moreover, FCM analysis results (Fig. 2I) showed that, compared to the control group, the expression of maturation markers CD86 and CD80 increased by 38.64% in the BL@OVA group and by 84.96% in the NGUPL@OVA group, whereas the Free OVA group exhibited negligible effects. Additionally, the BL@OVA group demonstrated a more pronounced promotion of BMDCs maturation compared to the Free OVA group, suggesting that the liposome-based delivery format may contribute to the enhanced efficacy [42]. Notably, the NGUPL group (without OVA) exhibited slightly better activation of BMDCs than the BL@OVA group, with the expression of maturation markers CD86 and CD80 increasing by 5.00%. Whereas the NGUPL@OVA group significantly enhanced BMDC maturation, showing a 33.41% increase in CD86/CD80 expression.

These results indicate that the intrinsic DC-activating property of NGUP serves as the primary factor in enhancing the immunostimulatory effects of the nanovaccine on immune cells. Both CLSM images (Fig. 2m) and flow cytometry data (Fig. 2k) further demonstrated that BMDCs treated with NGUPL@OVA expressed the highest level of SIINFEKL-H2K^b^ complex, 1.5-fold higher than the Free OVA group and 1.1-fold higher than the BL@OVA group. Taken together, as an adjuvant, NGUP enhances DCs uptake of OVA, promotes DCs maturation to strengthen antigen processing, and ultimately facilitates DCs-mediated antigen cross-presentation.

Normally, exogenous antigens activate CD4^+^T cells via MHC II, whereas cross-presentation enables exogenous antigens to also activate CD8^+^T cells through MHC I, thereby inducing tumor cell killing [43]. To evaluate the T-cell-activating capacity of the nanovaccine, we co-cultured NGUPL@OVA-stimulated BMDCs with splenic T lymphocytes isolated from C57 mice. CD69 is an early indicator of lymphocyte activation [44]. Consistent with the BMDCs maturation results, the percentage of CD69^+^T cells among CD8^+^T cells was highest in the NGUPL@OVA group, 1.4-fold higher than the Free OVA group, respectively (Fig. 2n). As well CFSE^+^T cells among CD8^+^T cells, achieving a level 1.7-fold higher than that in the Free OVA group (Fig. 2o). In addition, the crystal violet assay was performed to evaluate the enhancement of T cell-mediated tumor cell killing by NGUPL@OVA. After 24 h of co-culture with B16-OVA tumor cells, T cells in the NGUPL@OVA group exhibited the strongest tumor-killing efficacy, followed by the NGUPL group, which showed 1.8-fold greater cytotoxicity compared to the control (Fig. 2p and S11b). Lactate dehydrogenase (LDH) is primarily localized in the cytoplasm, but its release into the supernatant increases upon cell membrane damage [45]. Results revealed a significant elevation in LDH release in the NGUPL@OVA group, with a 64.48% increase compared to the control group (Fig. 2q). Furthermore, CD8^+^T cells secreted higher levels of the cytotoxic effector molecule granzyme B in the NGUPL@OVA group, which plays a pivotal role in immune-mediated tumor cell killing [46] (Fig. 2r). These results collectively demonstrate that NGUPL@OVA enhances T cell-dependent tumor cell killing by promoting DCs maturation.

### Enhanced lymph node biodistribution and prolonged residence of NGUPL@OVA

**Fig. 3:**
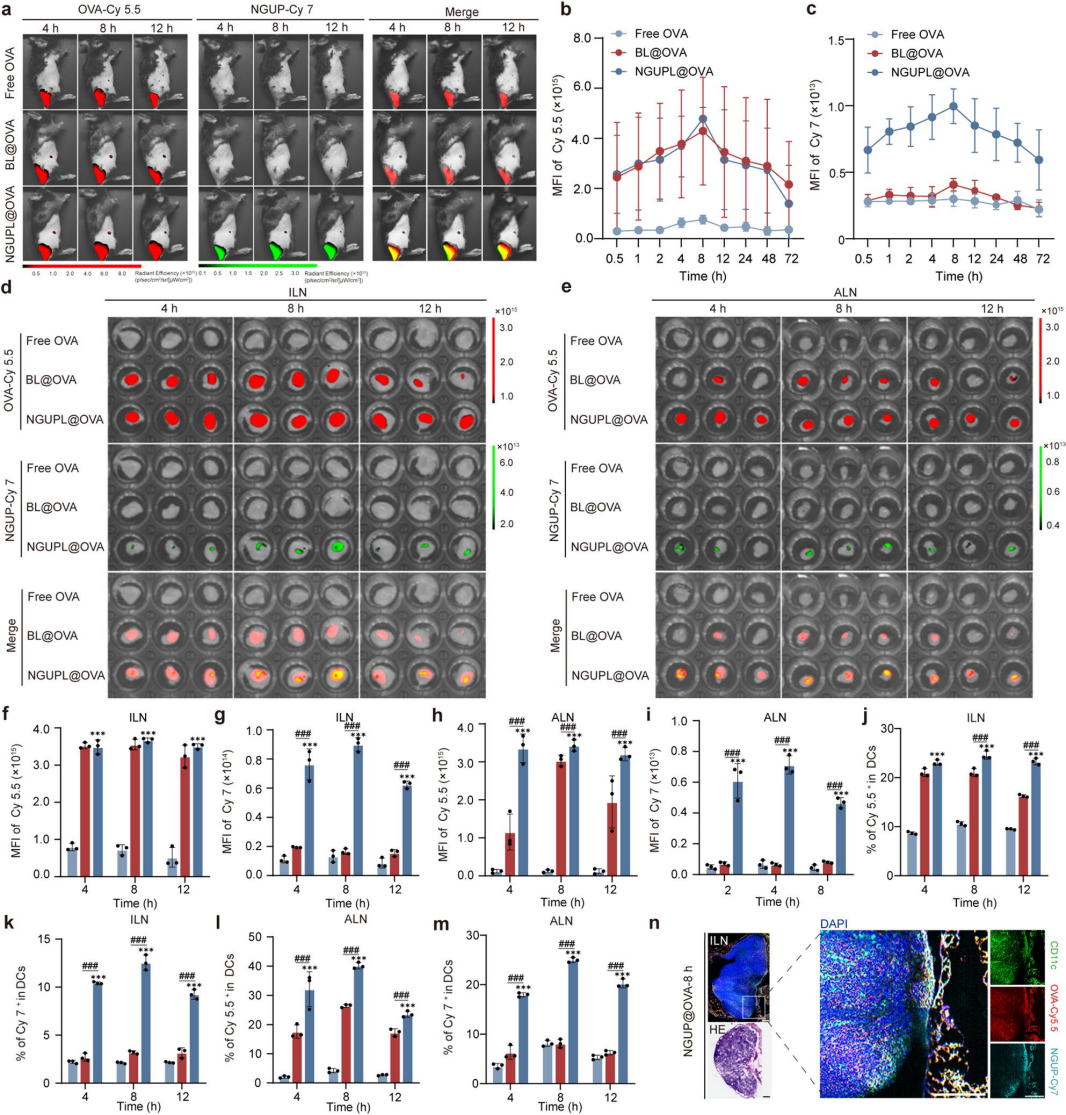
Enhanced lymph node biodistribution and prolonged residence of NGUPL@OVA. **a** In vivo biodistribution imaging of OVA-Cy5.5 and NGUP-Cy7 after intradermal injection in C57BL/6 mice at different time points using the IVIS Spectrum system. **b**, **c** Quantitative statistics of fluorescence signal from LNs in A). **d** Quantitative data of the Cy5.5 and Cy7 fluorescence signals of the ILNs collected at 2 h, 4 h, and 8 h after injection. **e** Quantitative data of the Cy5.5 and Cy7 fluorescence signal of the ALNs collected at 2 h, 4 h, and 8 h after injection. **f**, **g** Quantitative data of the Cy5.5 and Cy7 fluorescence signals of the ILNs collected at 2 h, 4 h, and 8 h after injection. **h**, **i** Quantitative data of the Cy5.5 and Cy7 fluorescence signals of the ALNs collected at 2 h, 4 h, and 8 h after injection. **j**, **k** The Cy5.5 and Cy7 in DCs of ILNs isolated from different treatment mice were detected and quantified by FCM. **l**, **m** The Cy5.5 and Cy7 in DCs of ALNs isolated from different treatment mice were detected and quantified by FCM. **n** Representative images of ILNs in mice treated by NGUP-Cy7 loading with OVA-Cy5.5 at 8 h following immunohistochemical staining for CD11c and H&E. The gating strategy for FCM is shown in Fig. S16. Data are shown as mean ± S.D. (*n* = 3). Statistical significance: *P < 0.05, ** *P* < 0.01 and *** *P* < 0.001 vs. Control; # *P* < 0.05, ## *P* < 0.01 and ### *P* < 0.001

The lymph node-targeting ability of tumor vaccines is crucial for their capacity to elicit effective anti-tumor immunity [47]. Hence, to assess the lymph node drainage ability of the NGUPL@OVA, C57BL/6 mice were intradermally injected with nanovaccines prepared from Cy5.5-labeled OVA and Cy7-labeled NGUP. The fluorescence intensity at the inguinal ILNs was measured using an IVIS at 30 min, 1 h, 2 h, 4 h, 8 h, 12 h, 24 h, 48 h, and 72 h post-injection (Fig. S13). The results showed that the OVA-Cy5.5 fluorescence intensity in ILNs gradually increased over time, peaking at 8 h. Compared to free OVA, NGUPL@OVA treatment induced a 4.3 -fold increase in fluorescence intensity, while exhibiting a level comparable to that of the BL@OVA group (Fig. 3a). This indicates that BL@OVA and NGUPL@OVA possess comparable lymph node targeting efficiency, suggesting that the improvement in delivery is primarily attributable to the liposomal formulation. Additionally, the fluorescence intensity trend of NGUP-Cy7 closely mirrored that of OVA-Cy5.5 (Fig. 3b-c). These findings demonstrate that NGUPL@OVA, with a size range of 20–200 nm, efficiently migrates from the administration site to the lymph nodes and exhibits prolonged retention. In addition, at 4 h, 8 h, and 12 h post-injection, the ILNs and distal ALNs were excised and imaged for fluorescence quantification (Fig. 3d-i). Both NGUP-Cy7 and OVA-Cy5.5 accumulated in the ILNs and ALNs. However, the fluorescence intensity of NGUPL@OVA in the ALNs was stronger than that of BL@OVA. This demonstrates that NGUPL@OVA can more effectively transport antigens to distal lymphoid tissues. FCM analysis results (Fig. 3j-m) revealed that, compared to BL@OVA, NGUPL@OVA exhibited a superior capacity to deliver antigens to BMDCs in the lymph nodes. Cryosectioned ILN samples visually confirmed the distribution and penetration depth of NGUPL@OVA within the lymph node tissue (Fig. 3n). Collectively, NGUPL@OVA can efficiently migrate to immune cell-rich LNs and specifically activate lymph node-resident DCs.

### NGUPL@OVA potentiates antigen-specific cellular immunity and establishes immune memory

**Fig. 4:**
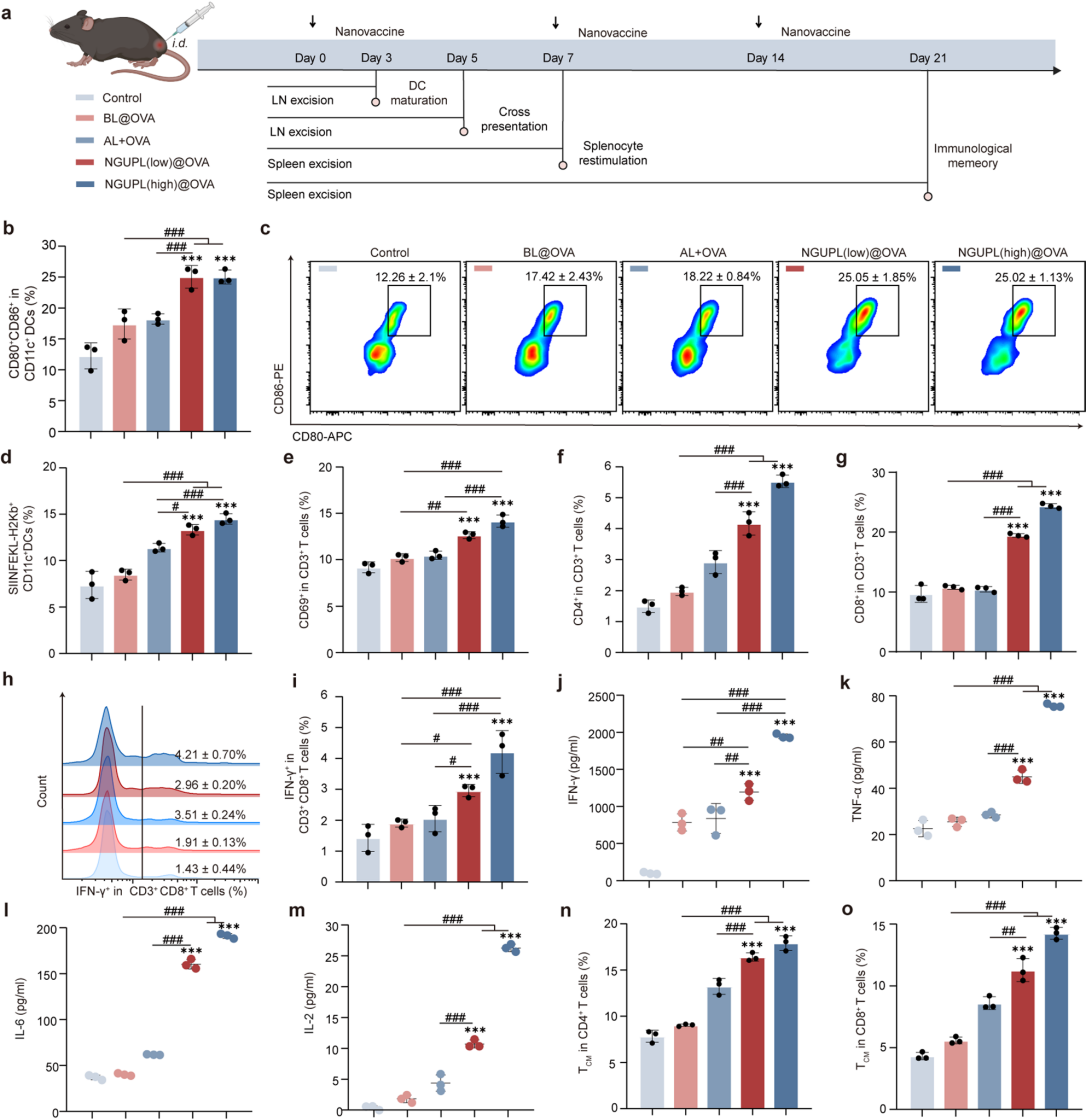
NGUPL@OVA potentiates antigen-specific cellular immunity and establishes immune memory in vivo. **a** The experimental design to evaluate the in vivo immune responses triggered by NGUPL@OVA nanovaccines. **b**, **c** Representative flow cytometry data and statistical data to show DC maturation induced by different formulations of nanovaccines in vivo on day 3 post-immunization. **d** Proportions of SIINFEKL-H2K^b+^ BMDCs in the LNs on day 5 post-immunization. **e** FCM analysis of the percentages of CD69^+^T lymphocytes. **f** FCM analysis of the percentages of CD4^+^T lymphocytes. **g** FCM analysis of the percentages of CD8^+^T lymphocytes. **h**, **i** IFN-γ-positive CD8^+^ T cells from restimulated splenocytes on day 7 after immunization. **j-m** The secretion of IFN-γ, TNF-α, IL-6 and IL-2 in the supernatants of splenocytes was measured by ELISA. **n**, **o** The expression of central memory cells in CD4^+^and CD8^+^ T cells was measured by FCM. The gating strategy for FCM is shown in Fig. S16. Data are shown as mean ± S.D. (*n* = 3). Statistical significance: *P < 0.05, ** *P* < 0.01 and *** *P* < 0.001 vs. Control; # *P* < 0.05, ## *P* < 0.01 and ### *P* < 0.001

The vaccination strategy illustrated in Fig. 4a was employed to evaluate the activation process of cellular immunity in vivo. By day 3 post-administration, the mature DC frequency in ILNs rose 43.65% and 37.31% higher in NGUPL@OVA-treated mice than BL@OVA group and AL + OVA group (Fig. 4b-c). On day 5 post-injection of NGUPL@OVA, FCM results demonstrated a marked elevation in the expression level of SIINFEKL-H2K^b^ on DCs within the ILNs (Fig. 4d). These findings indicate that NGUPL@OVA can promote DCs maturation, enabling mature DCs to efficiently process OVA, subsequently presenting the peptide-MHC complex (pMHC) on their surface for T cell recognition. This phenomenon aligns with the in vitro experimental results of NGUPL@OVA’s immunomodulatory effects on immune cells, confirming that NGUP functions as an effective vaccine adjuvant by activating DCs and enhancing antigen cross-presentation in vivo, with superior efficacy compared to aluminum adjuvant.

T cells specifically recognize the pMHC via their T cell receptors, thereby activating antigen-specific T cell responses and exerting effector functions, characterized by high efficiency, precision, and sustained effects [48]. A significant elevation in CD69⁺ and CD4⁺/CD8⁺T cell frequencies was observed in the NGUPL@OVA group versus controls (BL@OVA, AL + OVA) at day 7 post-vaccination, demonstrating its efficacy in facilitating DC-mediated T cell activation (Fig. 4e-g). Typically, stronger antigen-specific T-cell mediated immune responses occur on the second exposure to the same antigen [49]. During this process, T cells rapidly eliminate any cells that express this surface antigen by secreting IFN-γ. To investigate whether NGUPL@OVA triggered the OVA-specific T cell immune response, we measured their ability to induce the OVA-specific T cell response by restimulating the vaccinated mouse spleen cells in vitro co-incubated with OVA peptide and assaying the percentage of IFN-γ^+^CD8^+^T cells and the level of IFN-γ in cell supernatants on day 7. Compared to BL@OVA and AL + OVA controls, NGUPL@OVA significantly enhanced IFN-γ^+^CD8^+^T cells frequencies, inducing 2.8-fold and 2.2-fold increases in IFN-γ^+^CD8^+^T populations, respectively (Fig. 4h-i). Additionally, NGUPL@OVA stimulated T cells to secrete higher levels of cytokines (Fig. 4j-m), triggering a robust antigen-specific T cell immune response.

Memory T cells have long-term memory and specific recognition of antigens [50]. Establishing long-term immunological memory is the ultimate goal of vaccination. On day 21 post-vaccination, spleens were collected to evaluate whether the nanovaccine could induce immune memory formation. Compared to BL@OVA and AL + OVA, NGUPL@OVA significantly increased memory T cell proportions in both CD4^+^ and CD8^+^T cell populations. Specifically, CD4^+^ memory T cells rose by 98.34% and 35.36%, while CD8^+^ memory T cells increased by 155.04% and 65.58% versus BL@OVA and AL + OVA groups, respectively, indicating a strong immune memory response (Fig. 4n-o), with superior efficacy to aluminum adjuvant. In summary, NGUP, as an adjuvant, can indirectly enhance antigen-specific T cell responses by promoting DCs-mediated antigen uptake, processing, and MHC presentation.

### Prophylactic and therapeutic efficacy of NGUPL@OVA in B16-OVA melanoma-bearing mice

**Fig. 5:**
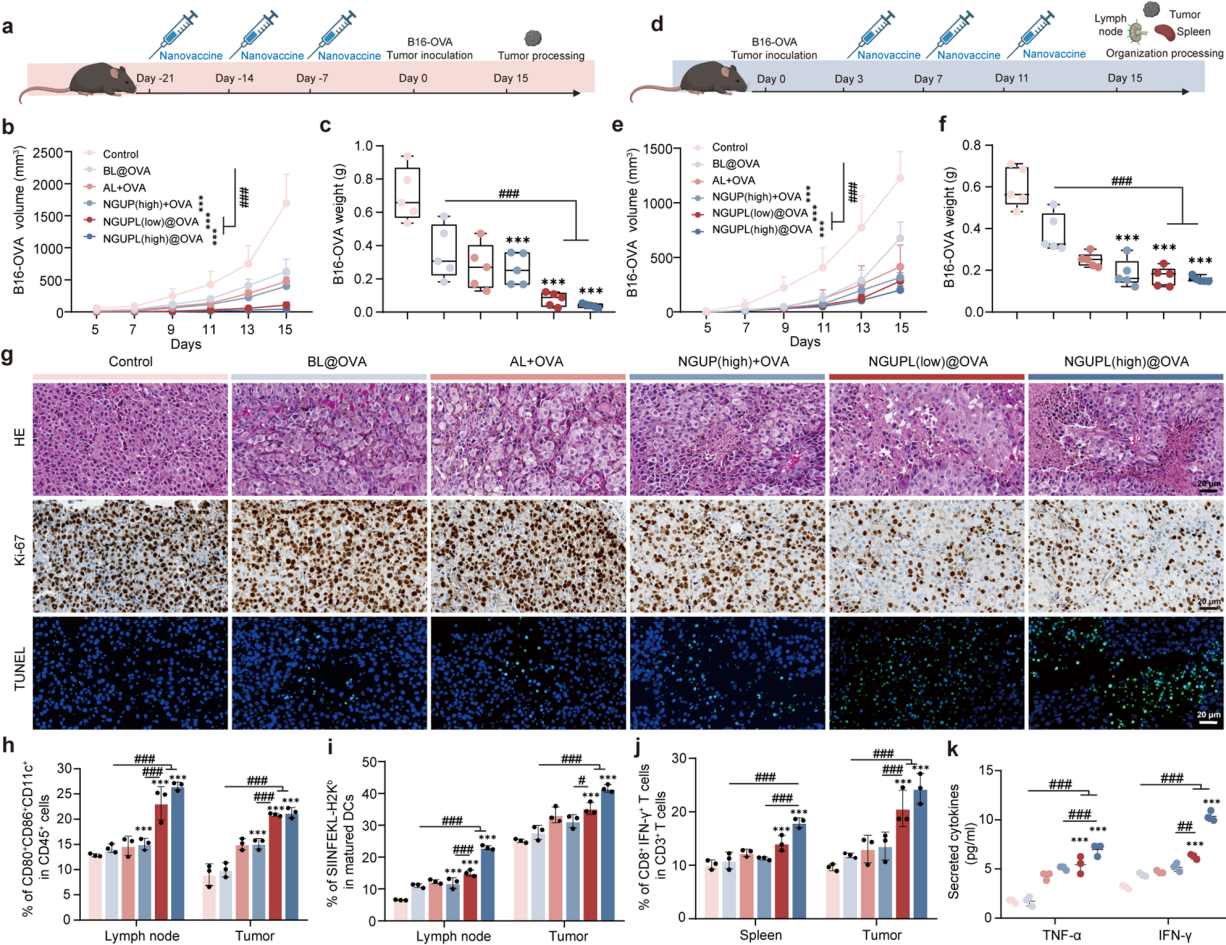
Prophylactic and therapeutic efficacy of NGUPL@OVA in B16-OVA melanoma-bearing mice. **a** Experimental timeline of drug administration in the prophylactic B16-OVA melanoma-bearing mice model. **b** Tumor growth kinetics of different groups of the prophylactic B16-OVA melanoma-bearing mice model (*n* = 5). **c** Tumor weight of different groups of the prophylactic B16-OVA melanoma-bearing mice model (*n* = 5). **d** Experimental timeline of drug administration in the therapeutic B16-OVA melanoma-bearing mice model. **e** Tumor growth kinetics of different groups of the therapeutic B16-OVA melanoma-bearing mice model. (*n* = 5). **f** Tumor weight of different groups of the therapeutic B16-OVA melanoma-bearing mice model (*n* = 5). **g** Representative images of H&E, Ki-67 and TUNEL staining were obtained from B16-OVA melanoma tumor samples collected from different groups (*n* = 3). **h** Percentages of mature DCs in LNs and tumor subjected to different treatments (gated on CD45^+^CD11c^+^) (*n* = 3). **i** Percentages of SIINFEKL-H2K^b+^ DCs in LNs and tumor subjected to different treatments (gated on CD45^+^CD11c^+^) (*n* = 3). **j** Percentages of CD8^+^IFN-γ^+^T in spleen subjected to different treatments (gated on CD45^+^CD11c^+^) (*n* = 3). **k** Levels of TNF-α and IFN-γ in tumor analyzed by ELISA (*n* = 3). The gating strategy for FCM is shown in Fig. S16. Data are shown as mean ± S.D. Statistical significance: *P < 0.05, ** *P* < 0.01 and *** *P* < 0.001 vs. Control; # *P* < 0.05, ## *P* < 0.01 and ### *P* < 0.001

After confirming that NGUPL@OVA could elicit robust adaptive immune responses in vivo, we further evaluated its prophylactic efficacy against murine melanoma. On day 7 after the final immunization, mice that had undergone three immunizations were challenged with B16-OVA melanoma tumor cells (Fig. 5a). The tumor growth kinetics (Fig. 5b), tumor weight (Fig. 5c) and gross tumor morphology (Fig. S14a) demonstrated that the PBS control group exhibited rapid tumor progression without any prophylactic effect. The BL@OVA, NGUP + OVA, and AL + OVA groups showed suboptimal preventive efficacy against murine melanoma. Strikingly, the NGUPL (high)@OVA group maintained significantly smaller mean tumor volumes at all measured timepoints, achieving superior prophylactic efficacy with a tumor inhibition rate of 97.10%. No significant body weight changes were observed during the experiment, indicating negligible toxicity of NGUPL@OVA (Fig. S14b). Additionally, TLR agonists as more modern adjuvants, demonstrate significant advantages in inducing antigen-specific T cell responses, which have a similar mechanism of action to NGUP. A comparative experiment was conducted to evaluate the effects of NGUP and the TLR9 agonist CpG ODN 1826 in mice bearing B16-OVA melanoma tumors. As shown in Fig. S15, the NGUP adjuvant demonstrated a level of melanoma prevention efficacy comparable to that of the CpG. These results further confirm that NGUP, as a novel adjuvant, not only outperforms the traditional aluminum adjuvant but also exhibits efficacy on par with modern adjuvants.

The therapeutic antitumor efficacy of NGUPL@OVA as a cancer vaccine was evaluated using a B16-OVA subcutaneous xenograft mouse model (Fig. 5d). Tumor growth curves (Fig. 5e) and excised tumor weights (Fig. 5f) demonstrated that the NGUPL (high)@OVA group exhibited optimal antitumor activity (tumor inhibition rate: 83.69%), with significantly superior efficacy compared to the BL@OVA and AL + OVA groups. Furthermore, to characterize tumor proliferation, apoptosis, and necrosis, excised tumor tissues were subjected to Ki-67 and TUNEL staining (Fig. 5g and S14d). Relative to the BL@OVA and AL + OVA groups, the NGUPL (high) @OVA group showed extensive necrosis and apoptosis, along with reduced tumor proliferation, indicating potent antitumor effects (Fig. S14c). The NGUPL (low) @OVA group displayed intermediate efficacy.

To investigate the mechanism of tumor growth inhibition, we sacrificed mice on day 15, Tumor-Draining Lymph Nodes (TDLNs), spleens and tumor were extracted to generate single-cell suspensions. First, DCs in TDLNs and Tumor were analyzed, and an obvious maturation effect of DCs in TDLNs was observed (Fig. 5h). The analysis of SIINFEKL-H2K^b^ in mature DCs revealed that NGUPL@OVA increased the expression of the pMHC on the surface of DCs (Fig. 5i). The observed tumor growth inhibition is typically closely associated with T cell infiltration. Further immunological analysis of T cell populations in both the spleen and tumor microenvironment demonstrated that NGUPL@OVA treatment significantly enhanced the generation of IFN-γ-secreting CD8^+^T cells, indicating the activation of a robust systemic anti-tumor immune response (Fig. 5j). Furthermore, a significant increase in the secretion of cytokines TNF-a and IFN-γ was detected in the tumor tissues of NGUPL@OVA groups (Fig. 5k). These results demonstrate that NGUP, as a vaccine adjuvant, can activate DCs, promote cross-presentation of antigens, and significantly enhance the infiltration of both CD8^+^T cells within the TME, thereby augmenting both prophylactic and therapeutic efficacy against murine melanoma.

### Universality of NGUP as a tumor vaccine adjuvant and safety evaluation

**Fig. 6:**
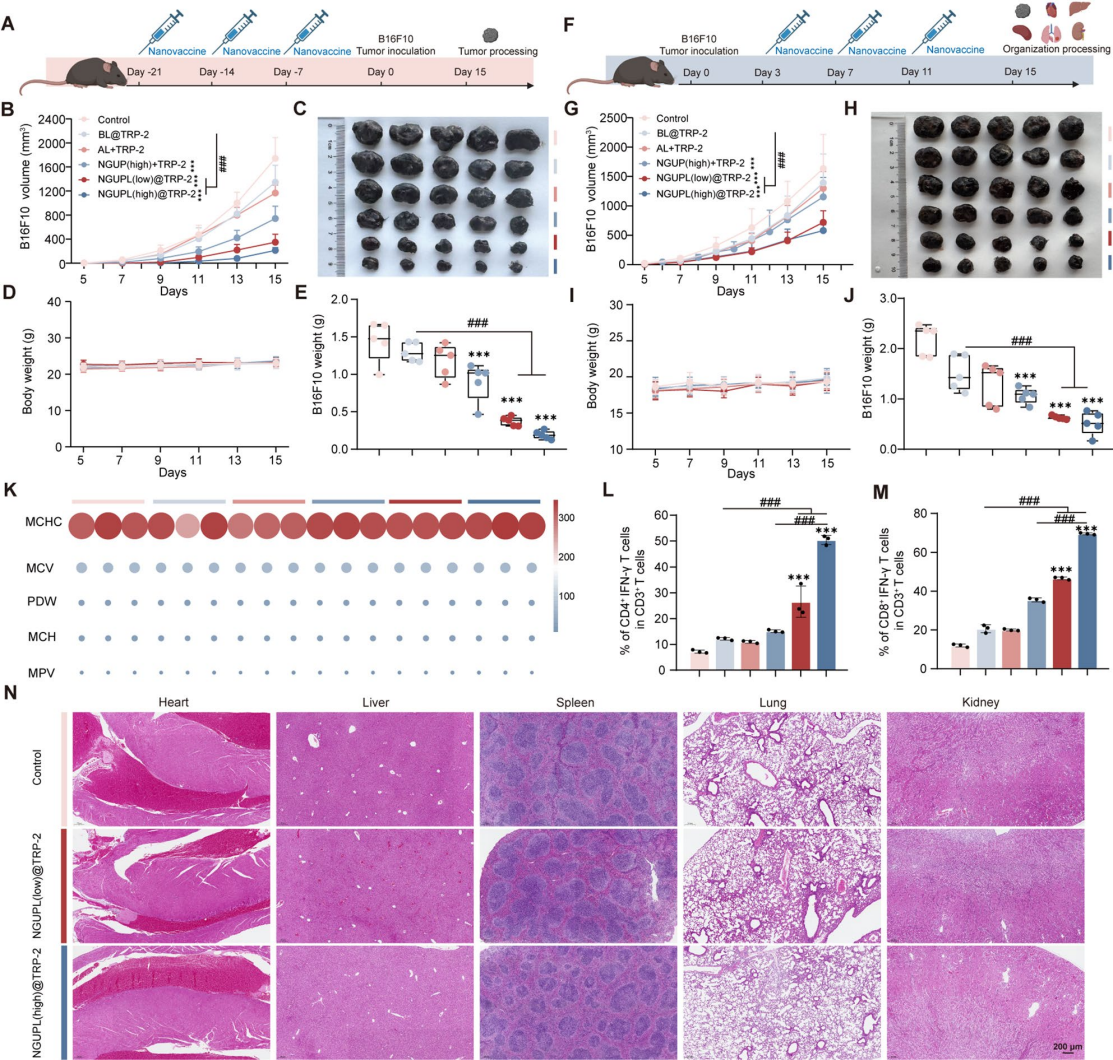
Evaluation of the universality and safety of NGUPL@OVA. **a** Experimental timeline of drug administration in the prophylactic B16F10 melanoma-bearing mice model. **b** Tumor growth kinetics of different groups of the prophylactic B16F10 melanoma-bearing mice model (*n* = 5). **c** Photograph of tumors from different groups of the prophylactic B16F10 melanoma-bearing mice model (*n* = 5). **d** Body weight of different groups of the prophylactic B16F10 melanoma-bearing mice model. **e** Tumor weight of different groups of the prophylactic B16F10 melanoma-bearing mice model. **f** Experimental timeline of drug administration in the therapeutic B16F10 melanoma-bearing mice model. **g** Tumor growth kinetics of different groups of the therapeutic B16F10 melanoma-bearing mice model (*n* = 5). **h** Photograph of tumors from different groups of the therapeutic B16F10 melanoma-bearing mice model (*n* = 5). **i** Body weight of different groups of the therapeutic B16F10 melanoma-bearing mice model. **j** Tumor weight of different groups of the therapeutic B16F10 melanoma-bearing mice model. **k** Full blood panel analysis performed on in vivo experiment mice (*n* = 3) (**l**) Percentages of CD4^+^IFN-γ^+^T in tumor subjected to different treatments (gated on CD45^+^CD11c^+^) (*n* = 5). **m** Percentages of CD8^+^IFN-γ^+^T in tumor subjected to different treatments (gated on CD45^+^CD11c^+^) (*n* = 5). **n** Representative images of H&E staining were obtained from mouse organs collected from different groups. The gating strategy for FCM is shown in Fig. S16. Data are shown as mean ± S.D. Statistical significance: *P < 0.05, ** *P* < 0.01 and *** *P* < 0.001 vs. Control; # *P* < 0.05, ## *P* < 0.01 and ### *P* < 0.001

The NGUPL@OVA nanovaccine constructed based on the OVA model antigen demonstrated significant antitumor effects in the B16-OVA tumor-bearing mouse model. To evaluate the versatility of the NGUP adjuvant, we selected the endogenous B16F10 melanoma antigen TRP-2 for substitution experiments. Similarly, the liposome co-encapsulation technique was employed to prepare the NGUPL@TRP-2 nanovaccine, and its preventive and therapeutic efficacy was assessed in the B16F10 tumor-bearing mouse model. In mice receiving the prophylactic vaccination regimen (Fig. 6a-e), the NGUPL (low)@TRP-2 and NGUPL (high)@TRP-2 groups exhibited the smallest tumors, with an inhibition rate reaching 87.82%. Moreover, the NGUP (high) + TRP-2 group showed significantly better preventive effects against tumors compared to the AL + TRP-2 group. These results indicate that NGUP, as a tumor vaccine adjuvant, has a remarkable prophylactic effect in the B16F10 tumor-bearing mouse model, outperforming aluminum adjuvant. In mice receiving the therapeutic vaccination regimen (Fig. 6f-n), the NGUPL (high)@TRP-2 group promoted the infiltration of IFN-γ^+^CD4^+^T cells and IFN-γ^+^CD8^+^T cells in the TME (Fig. 6l-m), demonstrating the best tumor suppression effect (inhibition rate:73.12%). The safety evaluation of NGUPL@TRP-2 included histological examination of major organs, no histological alterations were detected in the heart, liver, spleen, lungs, and kidney of the vaccinated mice (Fig. 6n). Furthermore, a comprehensive analysis of complete blood count showed no statistically significant differences among all groups (Fig. 6k). In summary, NGUP, as a tumor vaccine adjuvant, can effectively exert broad-spectrum antitumor immune effects. Its immunoenhancing capability is not dependent on a specific antigen type, and its prophylactic efficacy against tumors is generally superior to its therapeutic effects.

## Conclusion

We isolated a novel polysaccharide from Glycyrrhizae Radix Et Rhizoma named NGUP. We discovered that NGUP activates DCs via the TLR4/MyD88/TRAF6/NF-κB signaling pathway, thereby enhancing DCs-mediated cross-presentation of OVA.

Subsequently, we constructed a nanovaccine named NGUPL@OVA by encapsulating NGUP and OVA with lipid-based materials. In vitro, NGUPL@OVA enhances antigen-specific T cell responses indirectly by facilitating DC-mediated antigen uptake, processing, and MHC presentation. In vivo, NGUPL@OVA demonstrated efficient lymph node targeting capability, triggered robust cellular immune responses, and induced immunological memory. Notably, NGUP as a vaccine adjuvant showed superior efficacy in activating cellular immunity compared to aluminum adjuvants. Furthermore, NGUPL@OVA exhibited significant anti-tumor immune effects in both preventive and therapeutic models of B16-OVA murine melanoma. When TRP-2 was used to replace OVA in the preparation of NGUPL@TRP-2, it demonstrated excellent preventive and therapeutic efficacy against B16F10 melanoma, confirming the broad applicability of NGUP as a tumor vaccine adjuvant.

In summary, as a tumor vaccine adjuvant, NGUP not only exhibits excellent biocompatibility and safety but also effectively activates cellular immune responses by enhancing DCs-mediated antigen uptake and cross-presentation, thereby significantly potentiating T cell-dependent antitumor immunity. NGUP effectively overcomes the limitations of aluminum adjuvants, which induce weak cellular immunity and exhibit notable toxicity, demonstrating great potential as a novel adjuvant material. This study not only deepens our understanding of polysaccharide-mediated antitumor immunity but also provides foundational data for developing highly effective and low-toxicity tumor vaccine adjuvants.

## Supplementary Information

Supplementary Material 1.

## Data Availability

No datasets were generated or analysed during the current study.

## Abbreviations

NGUP: A novel polysaccharide from Glycyrrhizae Radix Et Rhizoma
BMDCs: Bone marrow derived dendritic cells
FBS: Fetal bovine serum
HPGPC: High-performance gel permeation chromatography
IC: Ion Chromatography
GC/MS: Methylation-gas chromatography-mass spectrometry
NMR: Nuclear magnetic resonance
TEM: Transmission electron microscope
DLS: Dynamic light scattering
LPS: Lipopolysaccharide
CLSM: Confocal laser scanning microscopy
ILNs: Inguinal Lymph Nodes
ALNs: Axillary Lymph Nodes
TME: Tumor microenvironment
pMHC: peptide-MHC complex
IFN-γ: Interferon-γ
TNF-α: Tumor necrosis factor
IL-6: Interleukin 6
TCM: Traditional Chinese Medicine
TLRs: Toll-like receptors
PAMPs: Pathogen-associated molecular patterns
