# Supplementary material for: Glycyrrhiza polysaccharide-adjuvanted liposomal vaccine potentiates tumor immunotherapy through lymph node-targeted modulation of the DC-T cell axis
Source: J Exp Clin Cancer Res. 2025 Nov 28;45:4. doi: 10.1186/s13046-025-03601-6 (PMC12764037; doi:10.1186/s13046-025-03601-6)
Supplement: Supplementary file 1 — Supplementary Material 1. [file 13046_2025_3601_MOESM1_ESM.docx]

**
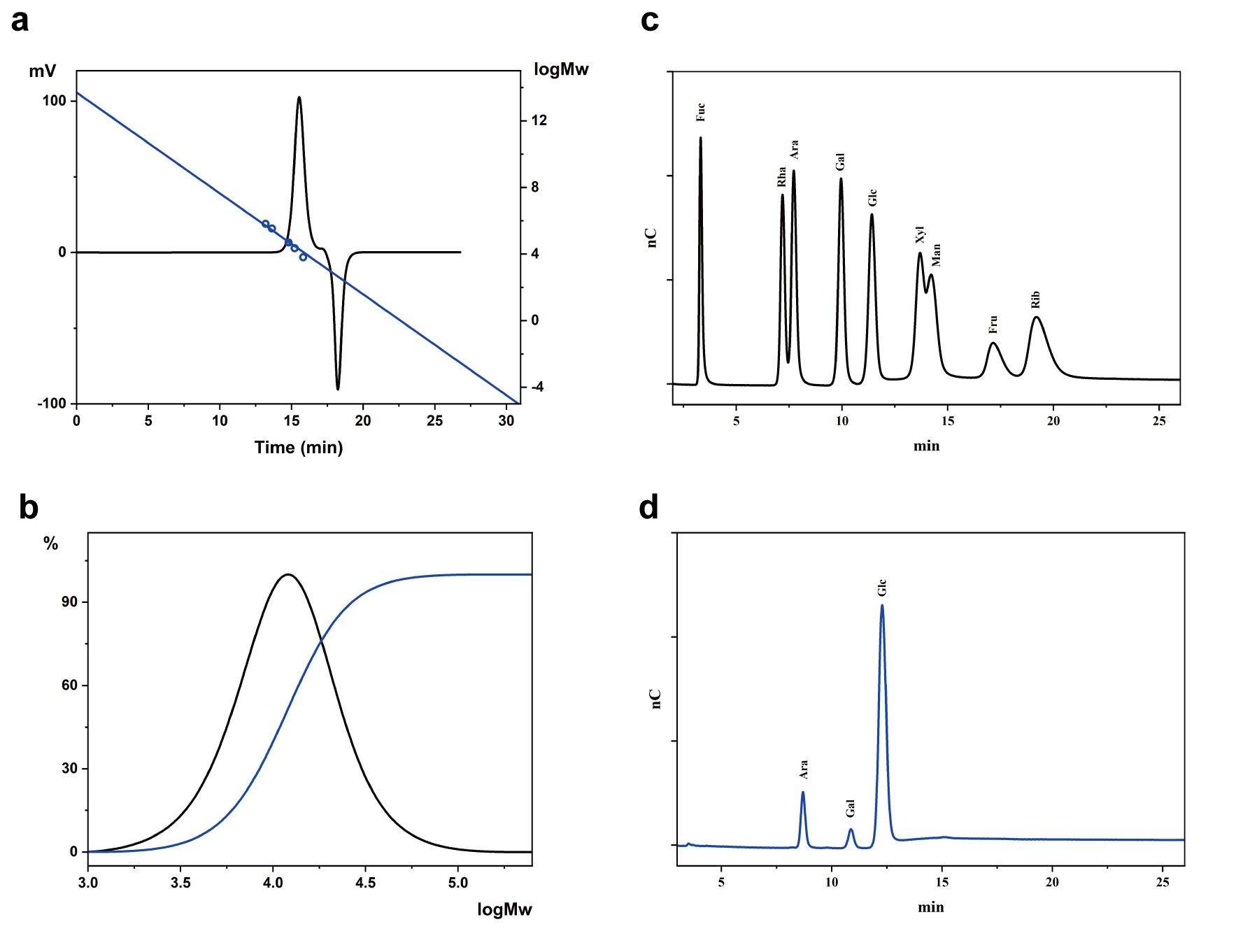
**

**Fig S1** Molecular weight and monosaccharide composition of NGUP. (**a-b**) Molecular weight of NGUP. (**c-d**) Monosaccharide composition of NGUP.

**
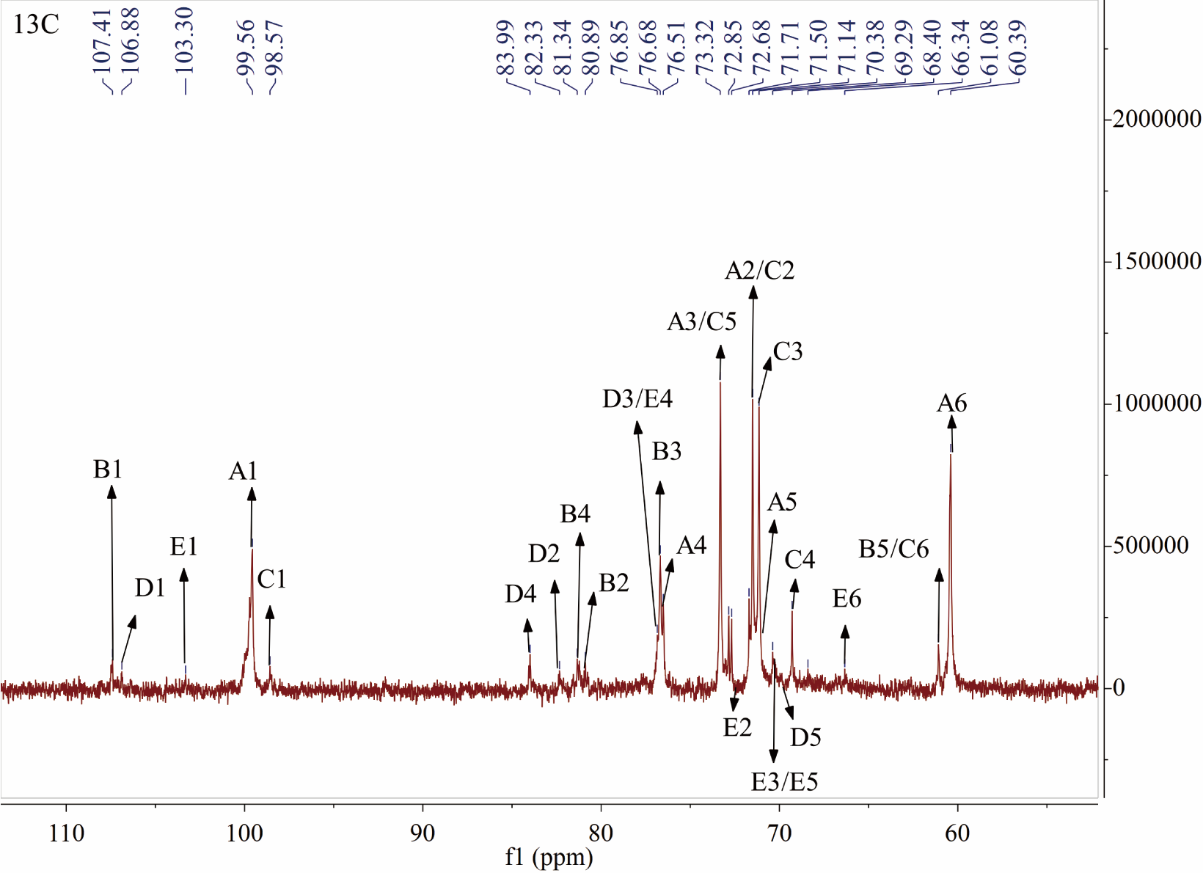
**

**Fig S2** ^13^C NMR spectrum.

**
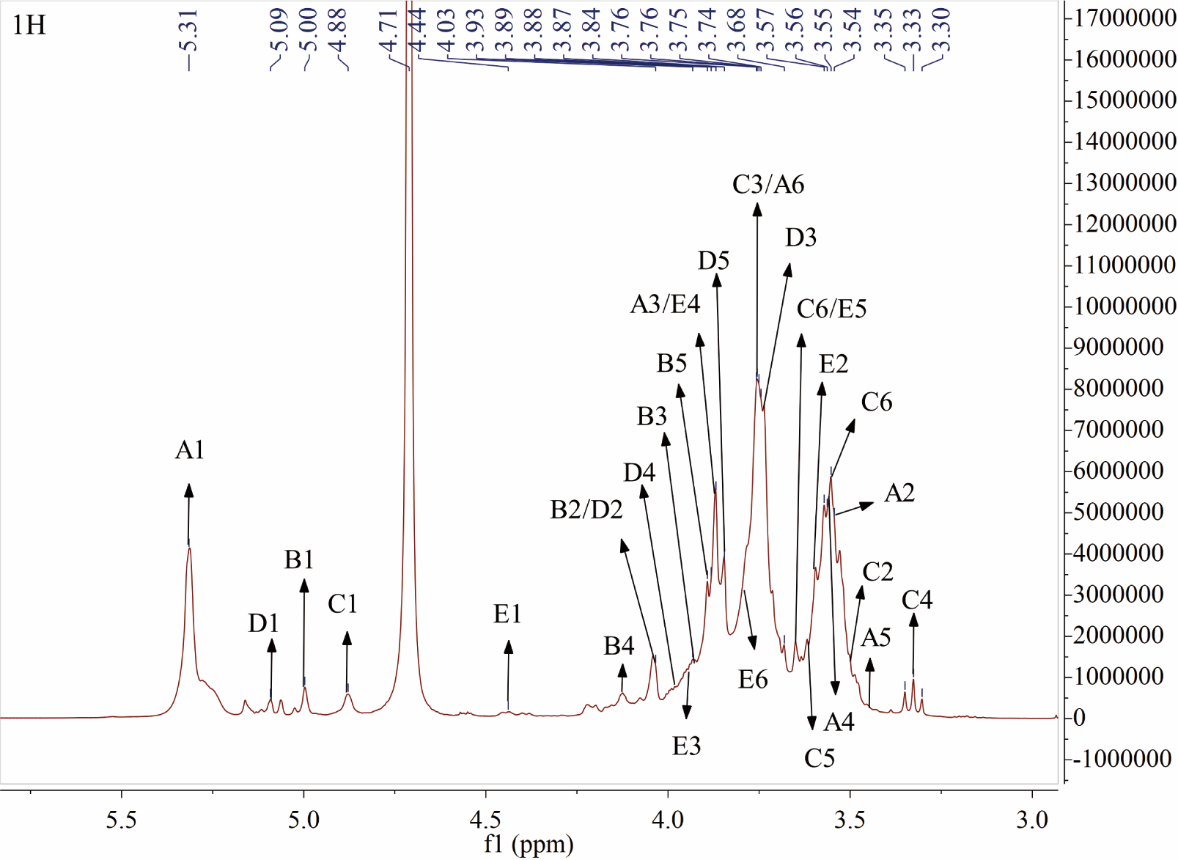
**

**Fig S3** ^1^H NMR spectrum.


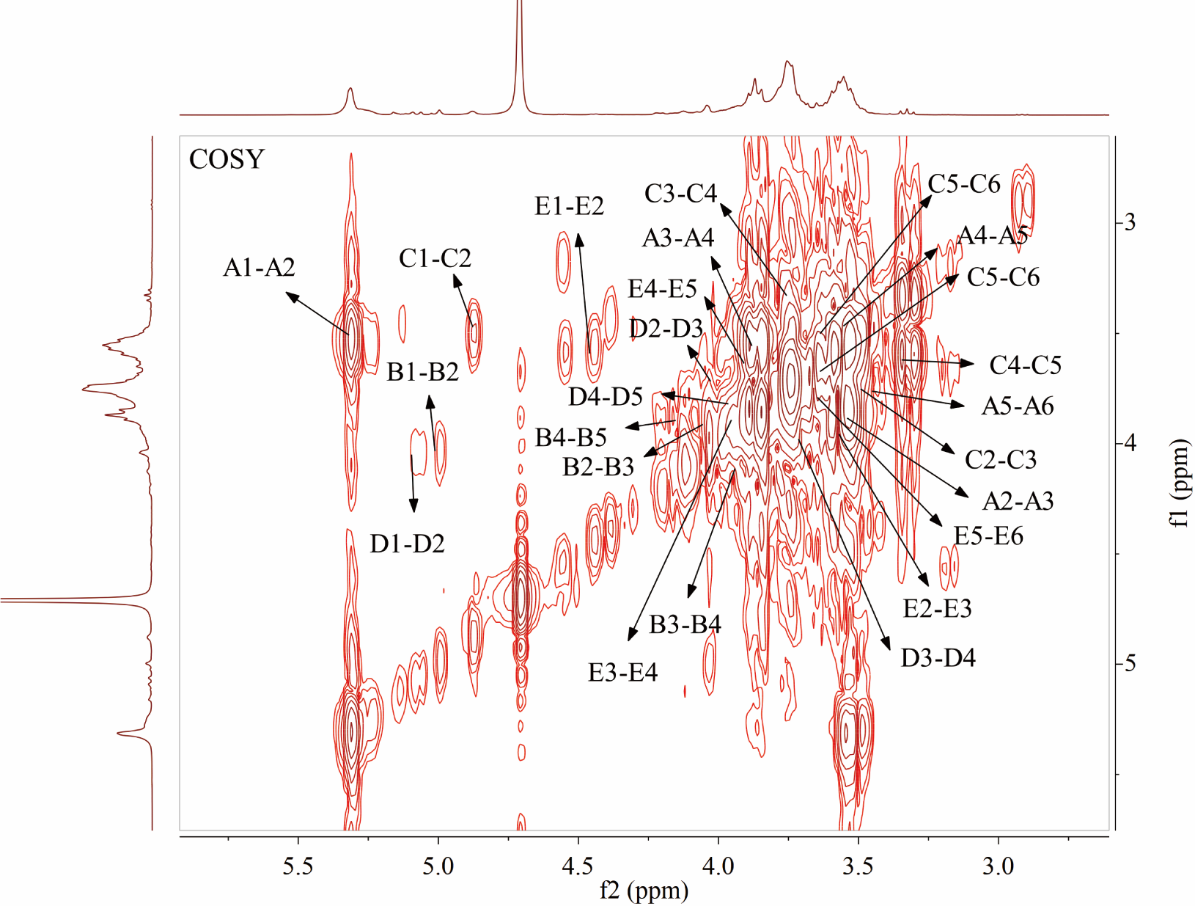


**Fig S4** ^1^H-^1^H COSY spectrum.

**
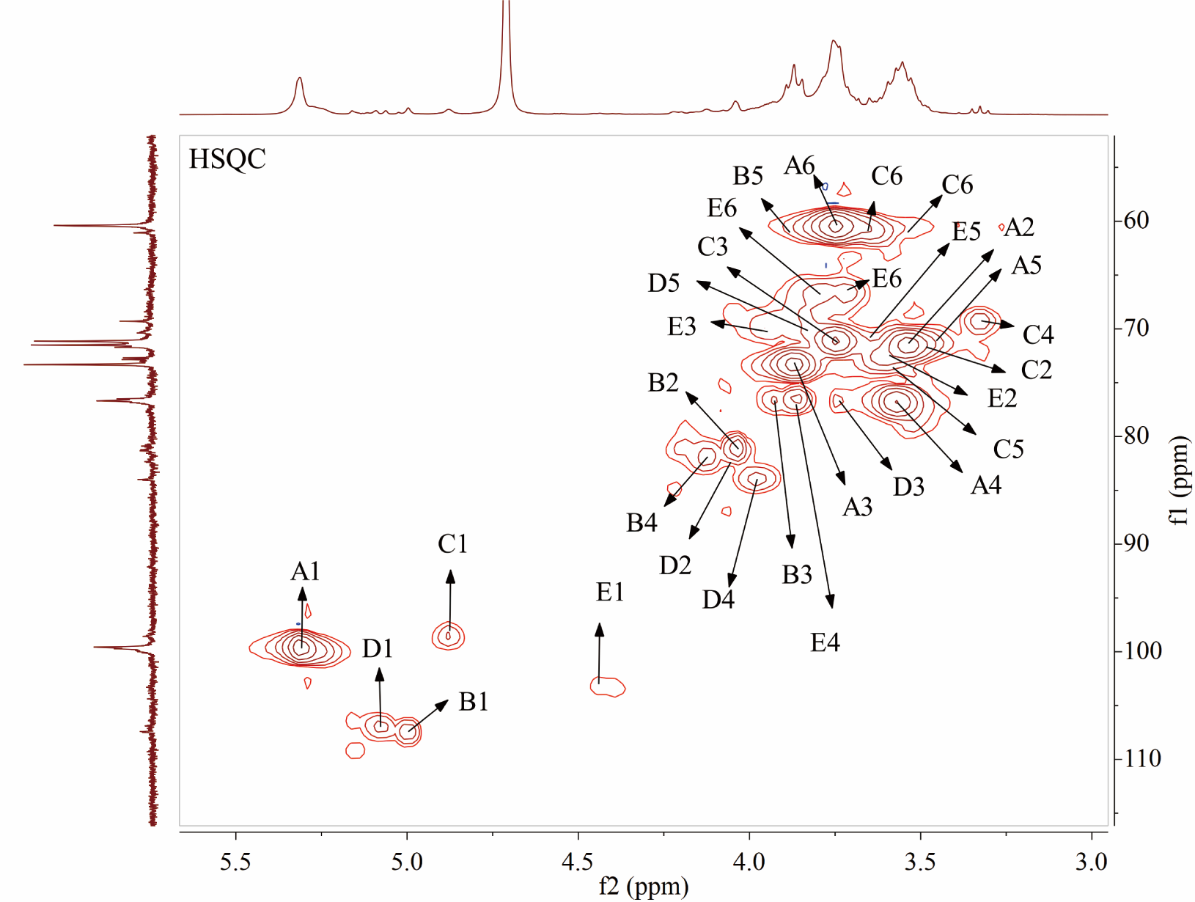
**

**Fig S5** HSQC spectrum.

**
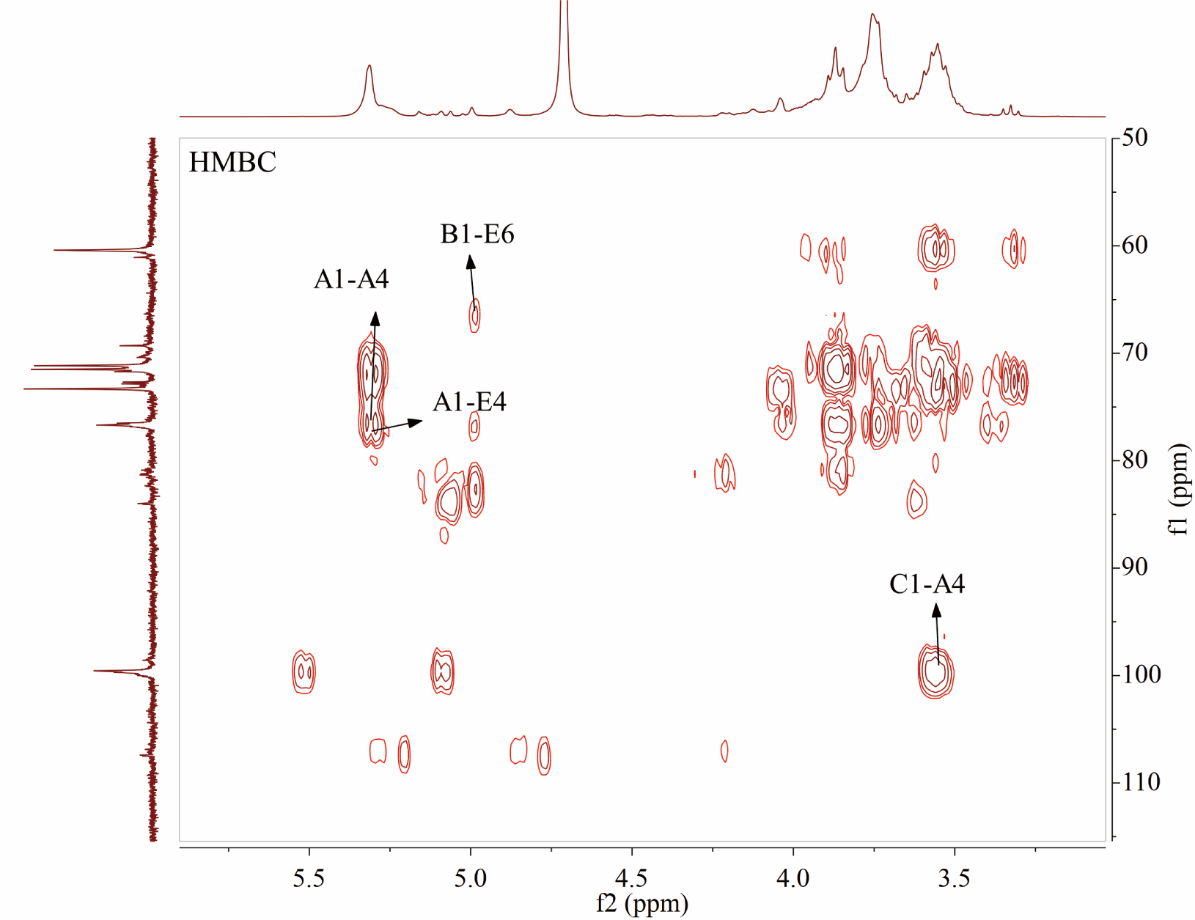
**

**Fig S6** HMBC spectrum.


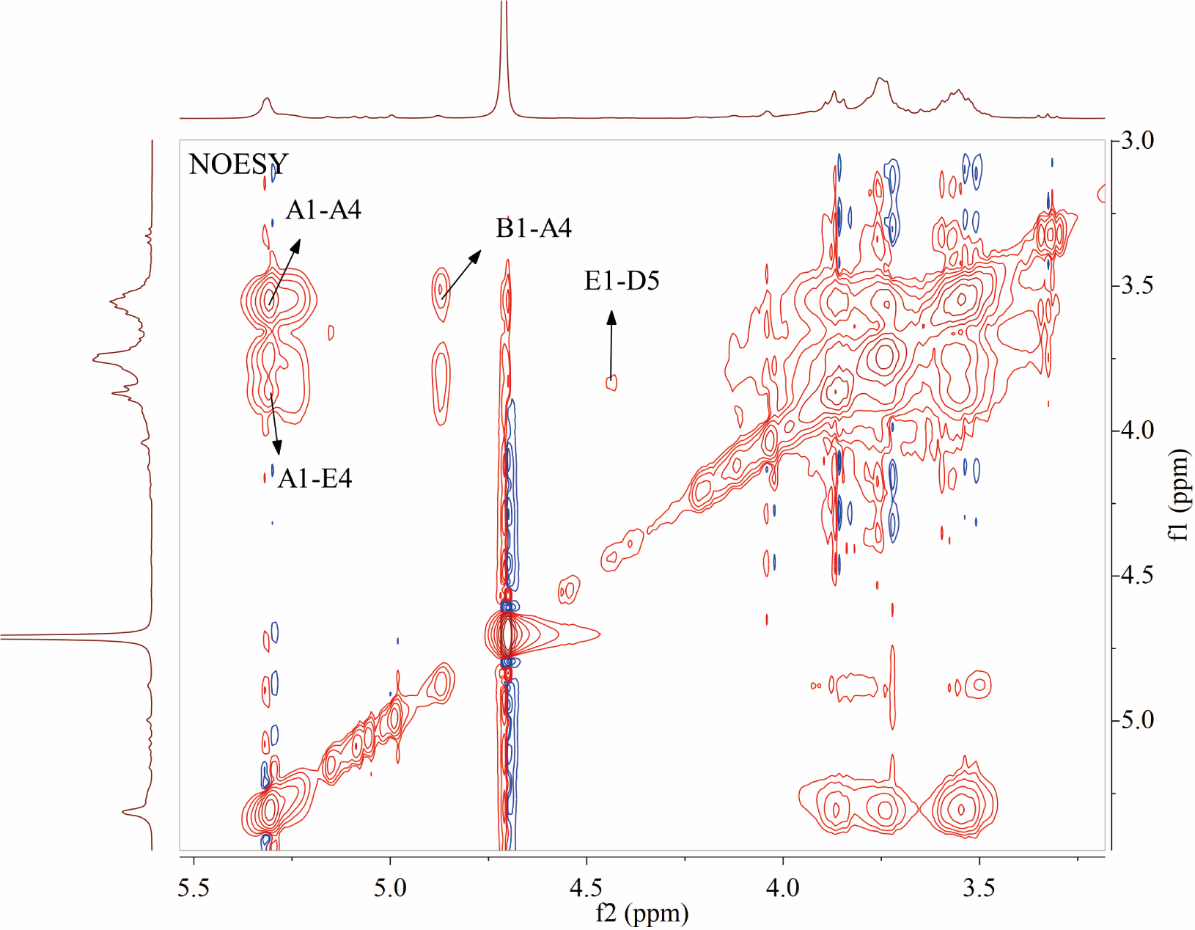


**Fig S7** NOESY spectrum.


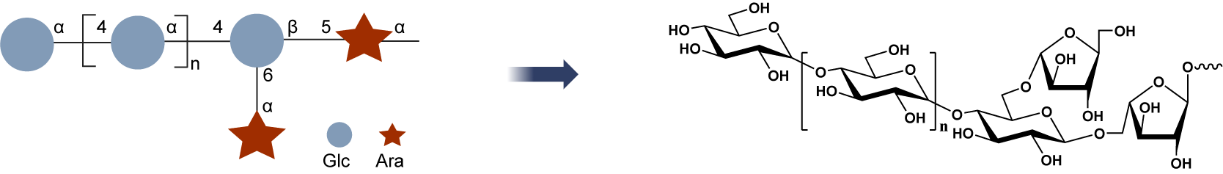


**Fig S8** The structure of NGUP.

**Table S1 Glycosidic residues analysis of NGUP by GC–MS.**

| Linking Method | Derivative name | RT | Relative molarity (%) |
| --- | --- | --- | --- |
| t-Ara(f) | 1,4-di-O-acetyl-2,3,5-tri-O-methyl arabinitol | 6.399 | 11.19 |
| t-Glc(p) | 1,5-di-O-acetyl-2,3,4,6-tetra-O-methyl glucitol | 9.435 | 8.77 |
| 5-Ara(f) | 1,4,5-tri-O-acetyl-2,3-di-O-methyl arabinitol | 11.183 | 7.11 |
| 4-Gal(p) | 1,4,5-tri-O-acetyl-2,3,6-tri-O-methyl galactitol | 13.541 | 3.40 |
| 4-Glc(p) | 1,4,5-tri-O-acetyl-2,3,6-tri-O-methyl glucitol | 14.722 | 64.69 |
| 3,4-Glc(p) | 1,3,4,5-tetra-O-acetyl-2,6-di-O-methyl glucitol | 16.882 | 1.24 |
| 4,6-Glc(p) | 1,4,5,6-tetra-O-acetyl-2,3-di-O-methyl glucitol | 18.996 | 3.61 |

**Table S2 ^1^H and ^13^C chemical shifts of NGUP.**

| Code | Glycosyl residues | Chemical shifts (ppm) | | | | | |
| --- | --- | --- | --- | --- | --- | --- | --- |
|  |  | H1/C1 | H2/C2 | H3/C3 | H4/C4 | H5/C5 | H6a,b/C6 |
| A | →4)-α-D-Glc*p*-(1→ | 5.31 | 3.52 | 3.87 | 3.56 | 3.44 | 3.75 |
|  |  | 99.56 | 71.5 | 73.32 | 76.51 | 70.88 | 60.39 |
| B | α-L-Ara*f*-(1→ | 5 | 4.04 | 3.93 | 4.13 | 3.89 | / |
|  |  | 107.41 | 80.89 | 76.68 | 81.34 | 61.09 | / |
| C | α-D-Glc*p*-(1→ | 4.88 | 3.49 | 3.75 | 3.33 | 3.62 | 3.54,3.66 |
| D |  | 98.57 | 71.48 | 71.14 | 69.28 | 73.06 | 61.08 |
|  | →5)-α-L-Ara*f*-(1→ | 5.09 | 4.04 | 3.73 | 3.98 | 3.83 | / |
|  |  | 106.88 | 82.33 | 76.85 | 83.99 | 69.87 | / |
| E | →4,6)-β-D-Glc*p*-(1→ | 4.44 | 3.59 | 3.95 | 3.87 | 3.65 | 3.79,3.71 |
|  |  | 103.3 | 72.46 | 70.1 | 76.78 | 70.28 | 66.34 |

**
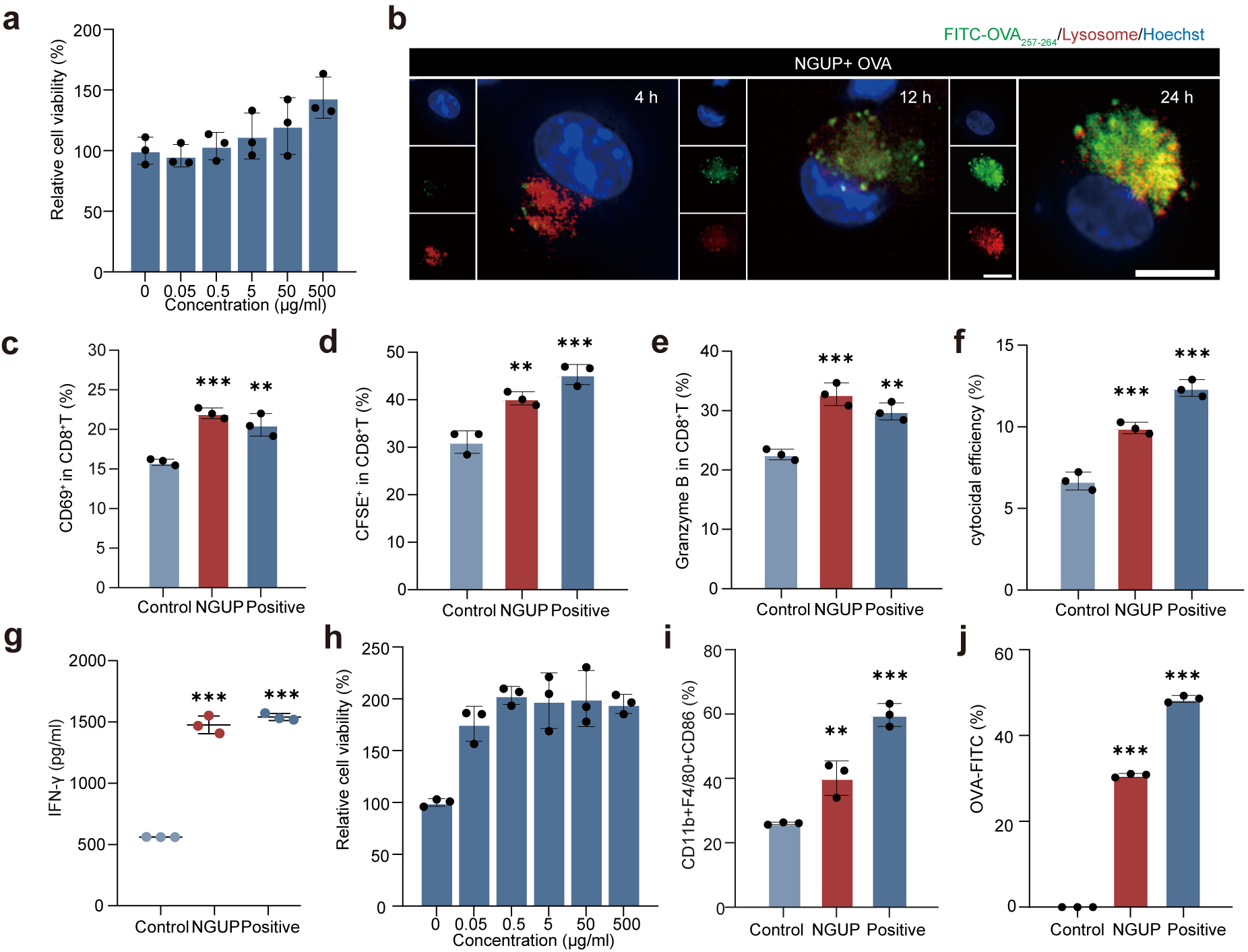
**

**Fig S9** (**a**) Cell viability of BMDCs after treatment with NGUP for 24 h. (**b**) CLSM images of antigen capture in BMDCs at 4, 12, or 24 h, respectively (Scale bars, 10 µm). (**c**) FCM analysis of the percentages of CD69^+^T lymphocytes in the co-incubation system.; (**d**) FCM analysis of the percentages of CFSE^+^T lymphocytes in the co-incubation system. (**e**) FCM analysis of the percentages of Granzyme B^+^T lymphocytes in the co-incubation system. (**f**) Quantification of tumor cell viability by crystal violet staining after 24 h co-culture with T cells. (**g**) FCM analysis of the percentages of IFN-γ^+^T lymphocytes in the co-incubation system. (**h**) Cell viability of BMDMs after treatment with NGUP for 24 h; (**i**) FCM analysis of the percentages of M1. (**j**) The intracellular uptake of FITC-OVA in BMDM cells was examined via FCM. Data are shown as mean ± S.D. (n = 3). Statistical significance: **P* < 0.05, ** *P* < 0.01 and *** *P* < 0.001 vs. Control; # *P* < 0.05, ## *P* < 0.01 and ### *P* < 0.001.

**
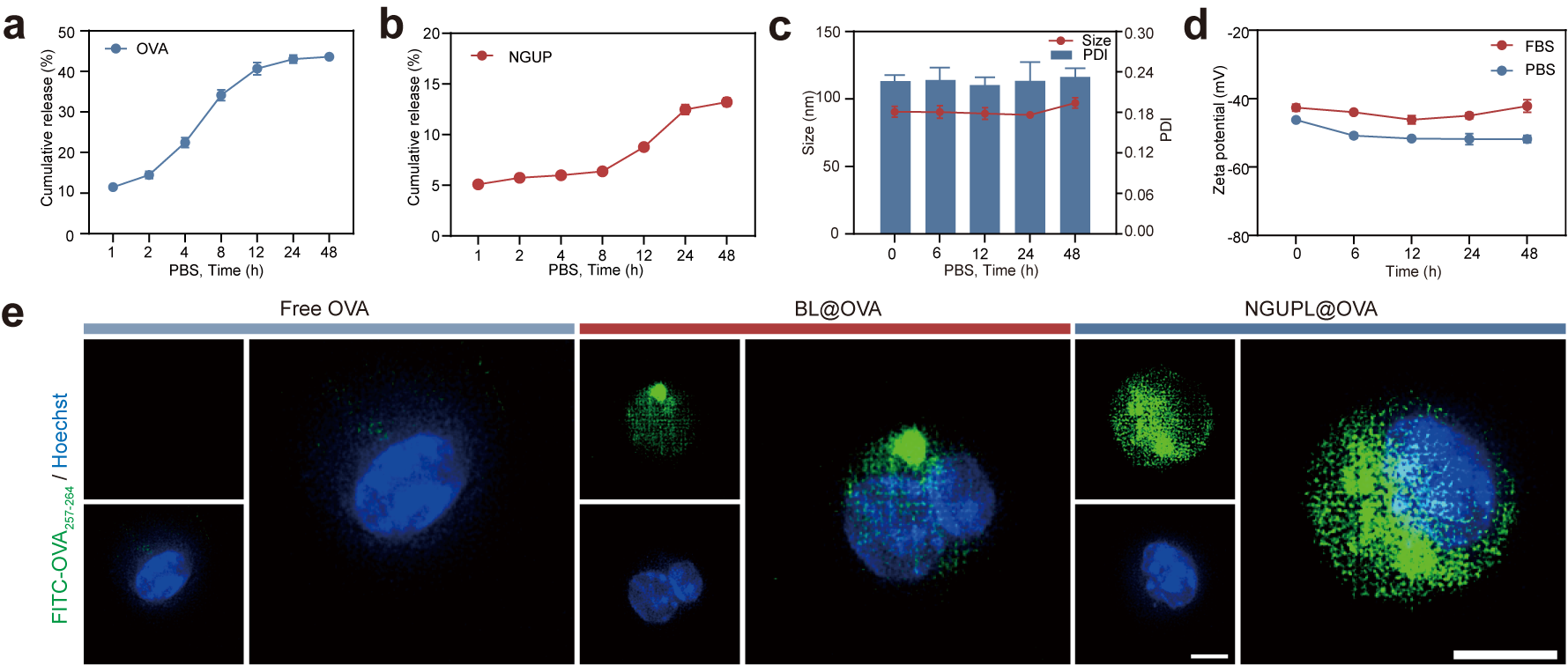
**

**Fig S10** Preparation and characterization of NGUPL@OVA. (**a**) OVA Release Curve. (**b**) NGUP Release Curve. (**c**) Size and PDI stability from aqueous solutions in pH 7.4 of NGUPL@OVA. (**d**) Zeta potentials stability from aqueous solutions in pH 7.4 and 10% FBS of NGUPL@OVA. (**e**) FITC-conjugated in BMDCs was detected and quantified via CLSM at 2h (Scale bars, 10 µm). Data are shown as mean ± S.D. (n = 3). Statistical significance: **P* < 0.05, ** *P* < 0.01 and *** *P* < 0.001 vs. Control; # *P* < 0.05, ## *P* < 0.01 and ### *P* < 0.001.

**
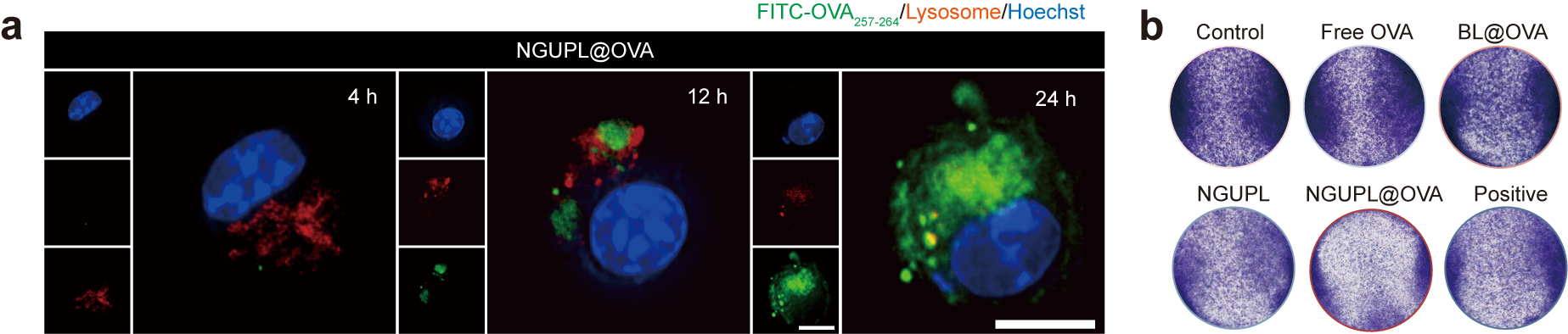
**

**Fig S11** (**a**) CLSM images of antigen capture in BMDCs at 4, 12, or 24 h, respectively (Scale bars, 10 µm). (**b**) Crystal violet staining image after co-culturing T cells with tumor cells. **
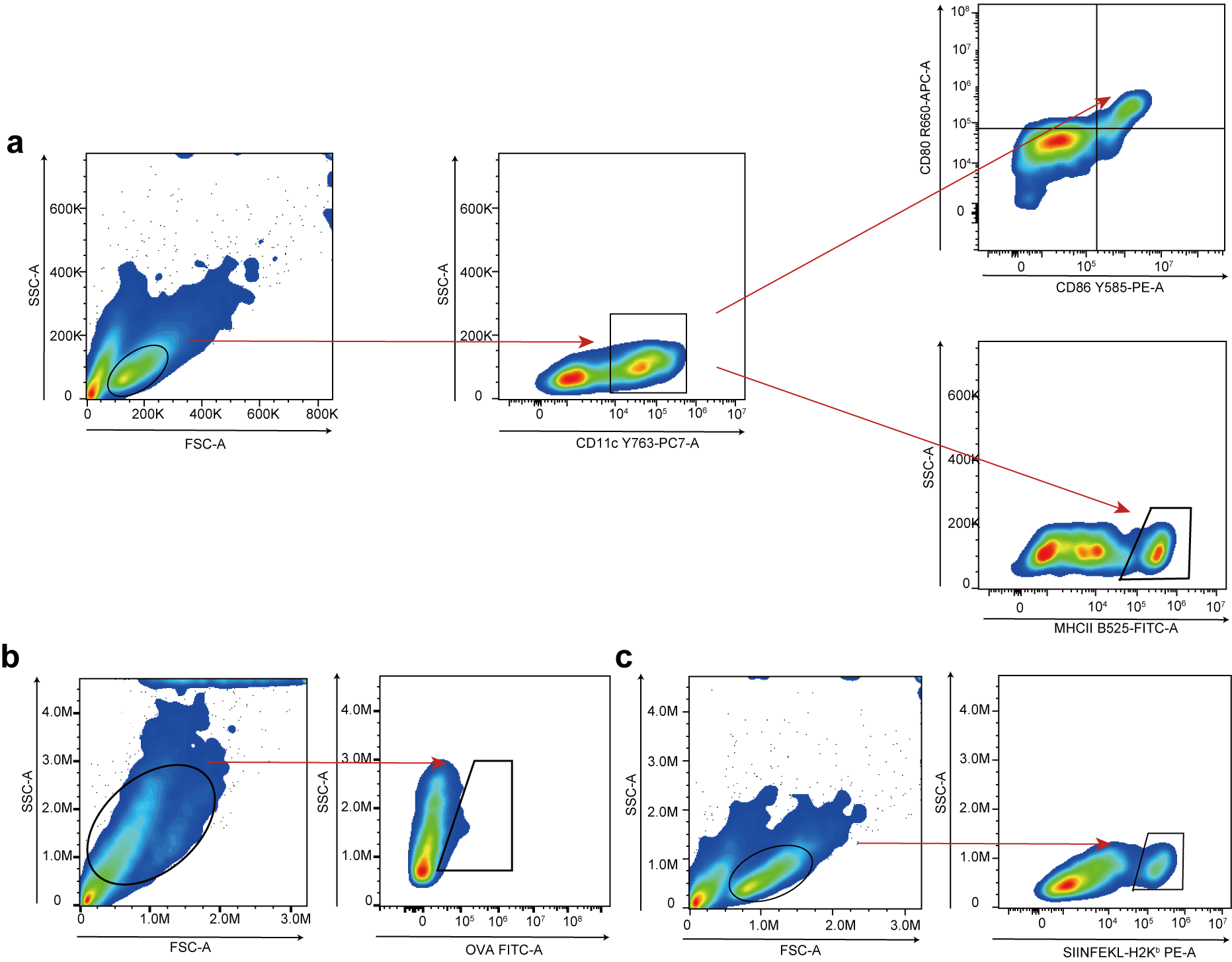
**

**Fig S12** Gating strategies used for FCM analysis of *in vitro* immune cells. (**a**) CD11C^+^ CD80^+^CD86^+^ or CD11C^+^MHCII^+^ BMDCs. (**b**) OVA-FITC^+^ BMDCs. (**c**) SIINFEKL-H2K^b+^ BMDCs.

**
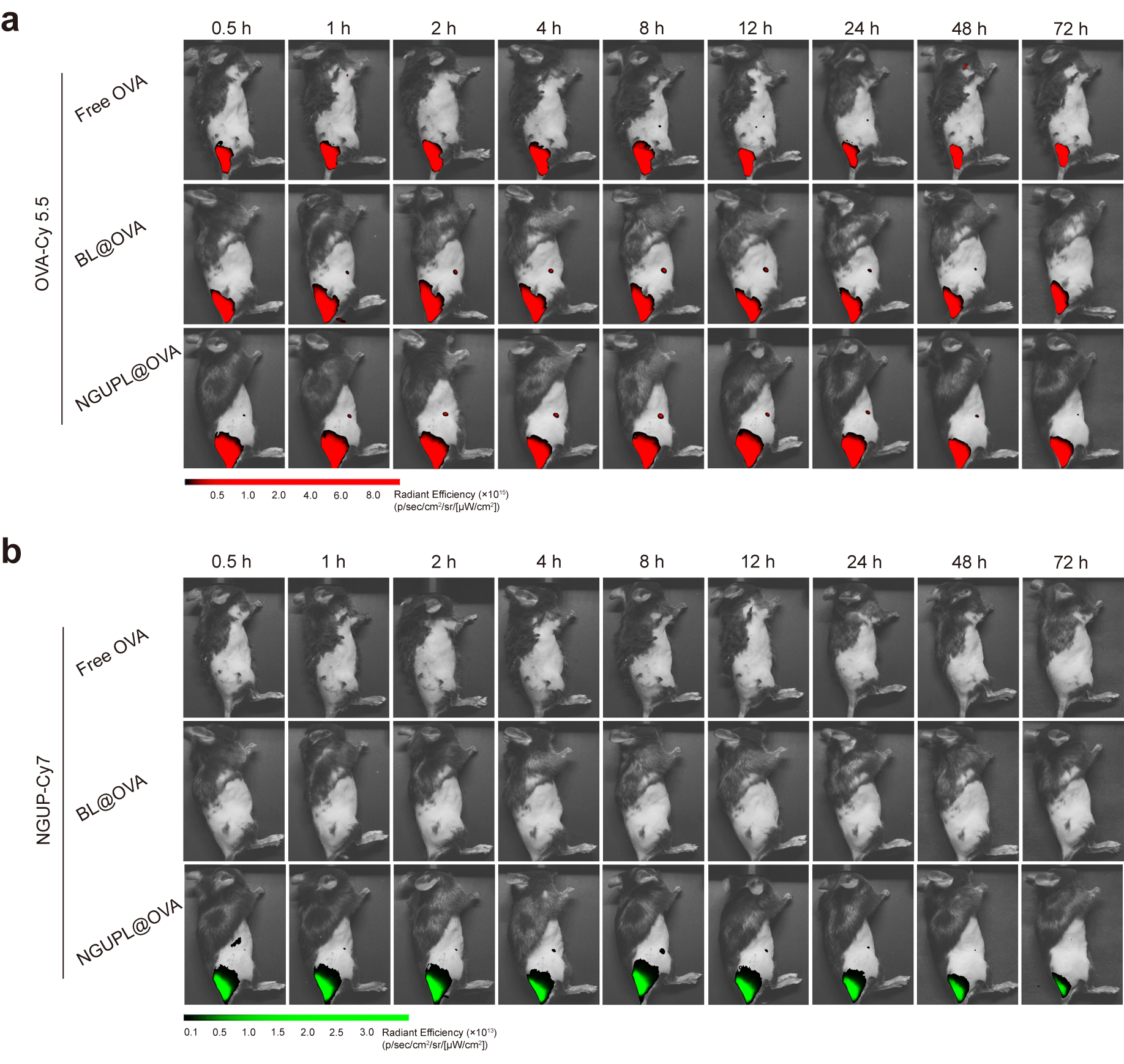
**

**Fig S13** NGUPL@OVA nanovaccines delivery and antigen presentation capabilities. (**a**) *In vivo* biodistribution imaging of OVA-Cy5.5 after intradermal injection in C57BL/6 mice at different time points using the IVIS Spectrum system. (**b**) *In vivo* biodistribution imaging of NGUP-Cy7 after intradermal injection in C57BL/6 mice at different time points using the IVIS Spectrum system. Data are mean ± SD, n = 3.


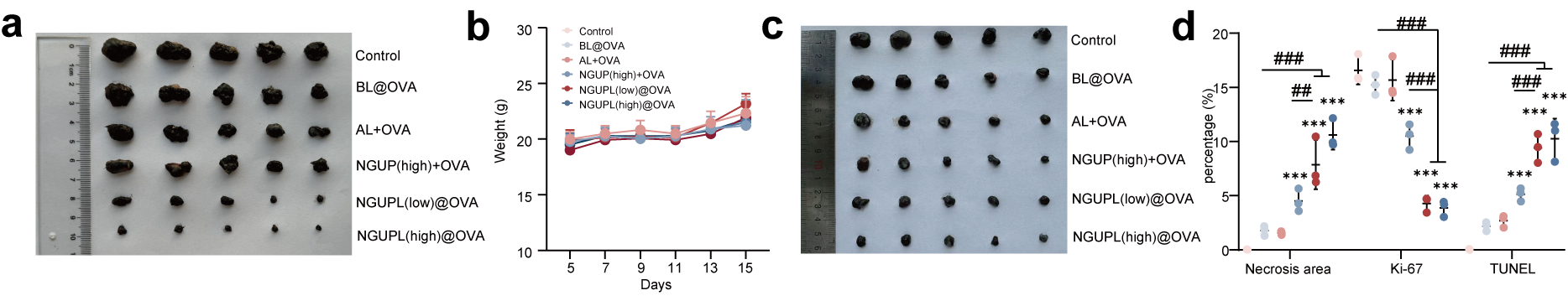


**Fig S14** Tumor suppression efficacy of the NGUPL@OVA in mice bearing B16-OVA tumours. (**a**) Tumor Image of different groups of the prophylactic B16-OVA melanoma-bearing mice model (n=5). (**b**) Body weight of different groups of the prophylactic B16-OVA melanoma-bearing mice model (n=5). (**c**) Tumor Image of different groups of the therapeutic B16-OVA melanoma-bearing mice model (n=5). (**d**) Quantification of H&E, Ki-67, and TUNEL staining regions in tumour samples collected from different groups on day 16 (n=3). Data are mean ± SD, n = 3; **P* < 0.05, ** *P* < 0.01 and *** *P* < 0.001 vs. Control; # *P* < 0.05, ## *P* < 0.01 and ### *P* < 0.001.


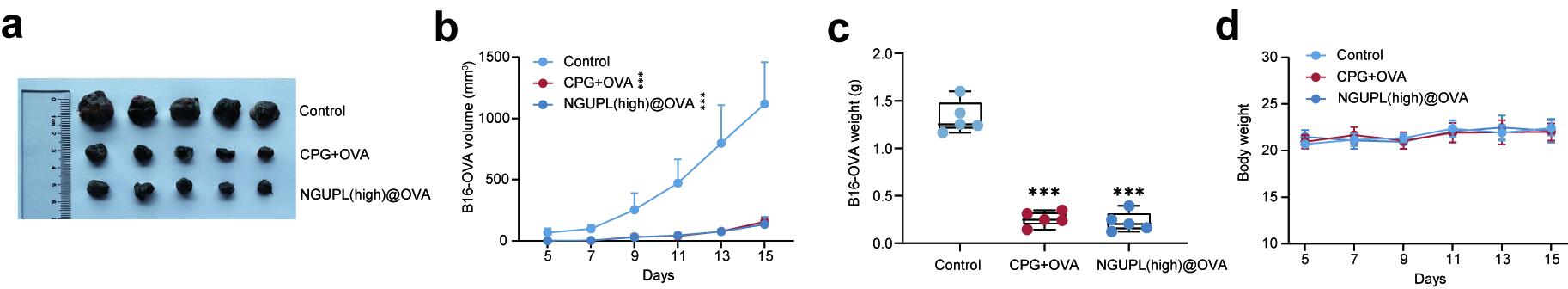


**Fig S15** (**a**) Tumor Image of different groups of the prophylactic B16-OVA melanoma-bearing mice model (n=5). (**b**) Tumor growth kinetics of different groups of the prophylactic B16-OVA melanoma-bearing mice model (n=5). (**c**) Tumor weight of different groups of the prophylactic B16-OVA melanoma-bearing mice model (n=5). (**d**) Body weight of different groups of the prophylactic B16-OVA melanoma-bearing mice (n=5). Data are mean ± SD, **P* < 0.05, ** *P* < 0.01 and *** *P* < 0.001 vs. Control.

**
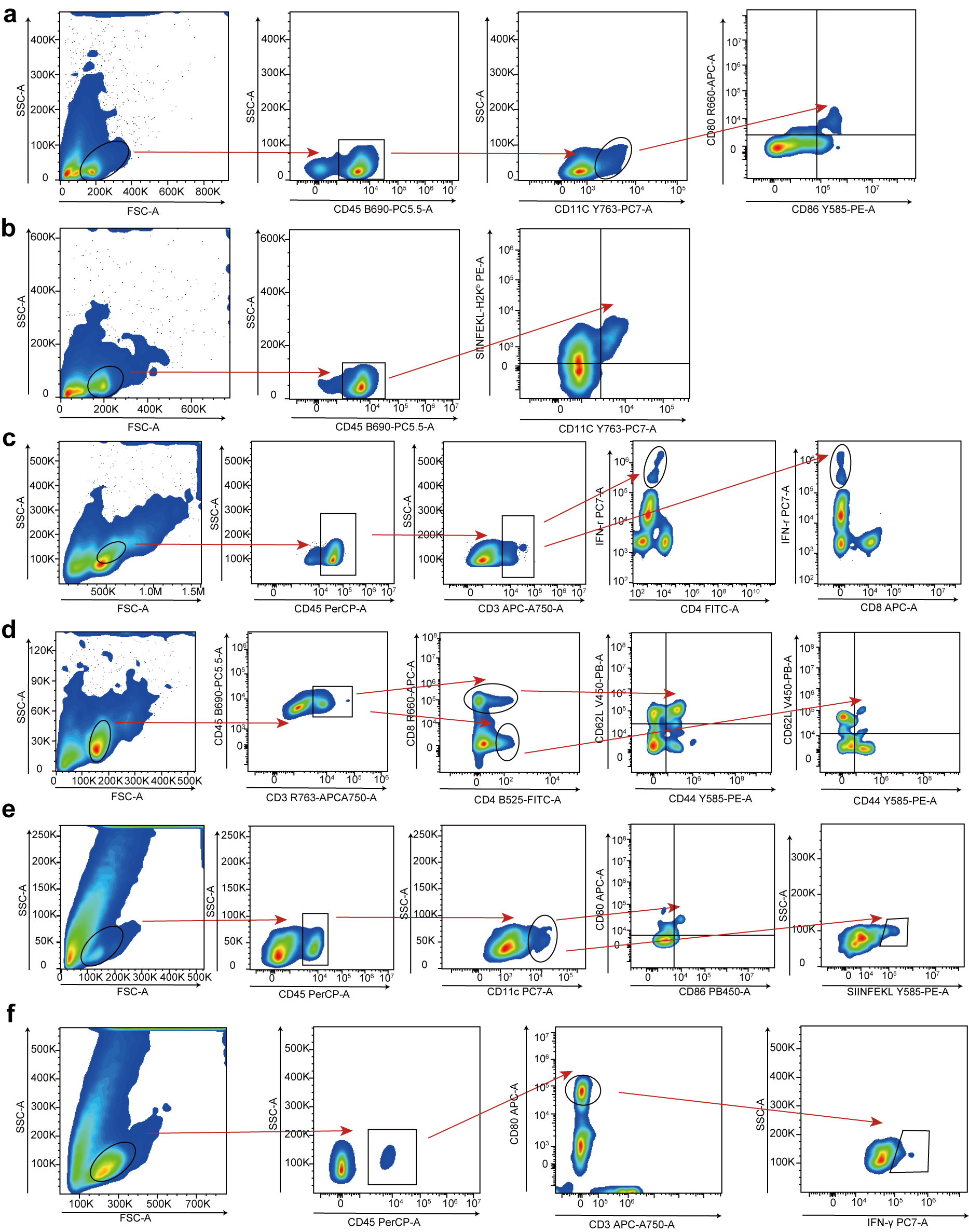
**

**Fig S16** Gating strategies used for FCM analysis of *in vivo* immune cells. (**a**) CD45^+^CD11C^+^ CD80^+^CD86^+^ in lymph nodes. (**b**) CD45^+^CD11C^+^ SIINFEKL-H2K^b+^ in lymph nodes. (**c**) CD45^+^CD3^+^CD4^+^ and CD45^+^CD3^+^CD8^+^ T cells in spleen. (**d**) CD45^+^CD3^+^CD4^+^ CD44^+^CD62L^+^ and CD45^+^CD3^+^CD8^+^ CD44^+^CD62L^+^ T cells in spleen. (**e**) CD45^+^CD11C^+^ CD80^+^CD86^+^ and CD45^+^CD11C^+^ SIINFEKL-H2K^b+^ in tumours. (**f**) CD45^+^CD3^+^CD8^+^ T cells in tumours.
